# Global dynamics and computational modeling for analyzing and controlling Hepatitis B: A novel epidemic approach

**DOI:** 10.1371/journal.pone.0304375

**Published:** 2024-06-27

**Authors:** Muhammad Farhan, Zahir Shah, Zhi Ling, Kamal Shah, Thabet Abdeljawad, Saeed Islam, Hakim A. L. Garalleh

**Affiliations:** 1 School of Mathematical Science, Yangzhou University, Yangzhou, China; 2 Department of Mathematical Sciences, University of Lakki Marwat, Lakki Marwat, KPK, Pakistan; 3 Department of Mathematics and Sciences, Prince Sultan University, Riyadh, Saudi Arabia; 4 Department of Computer Science and Mathematics Lebanese American University, Byblos, Lebanon; 5 Department of Mathematics, Abdul Wali Khan University Mardan, Mardan, Khyber Pakhtunkhwa, Pakistan; 6 Department of Mathematical Science, College of Engineering, University of Business and Technology, Jeddah, Saudi Arabia; Centers for Disease Control and Prevention, UNITED STATES

## Abstract

Hepatitis B virus (HBV) infection is a global public health issue. We offer a comprehensive analysis of the dynamics of HBV, which can be successfully controlled with vaccine and treatment. Hepatitis B virus (HBV) causes a significantly more severe and protracted disease compared to hepatitis A. While it initially presents as an acute disease, in approximately 5 to 10% of cases, it can develop into a chronic disease that causes permanent damage to the liver. The hepatitis B virus can remain active outside the body for at least seven days. If the virus penetrates an individual’s body without immunization, it may still result in infection. Upon exposure to HBV, the symptoms often last for a duration ranging from 10 days to 6 months. In this study, we developed a new model for Hepatitis B Virus (HBV) that includes asymptomatic carriers, vaccination, and treatment classes to gain a comprehensive knowledge of HBV dynamics. The basic reproduction number $R_{0}$ is calculated to identify future recurrence. The local and global stabilities of the proposed model are evaluated for values of $R_{0}$ that are both below and above 1. The Lyapunov function is employed to ensure the global stability of the HBV model. Further, the existence and uniqueness of the proposed model are demonstrated. To look at the solution of the proposed model graphically, we used a useful numerical strategy, such as the non-standard finite difference method, to obtain more thorough numerical findings for the parameters that have a significant impact on disease elimination. In addition, the study of treatment class in the population, we may assess the effectiveness of alternative medicines to treat infected populations can be determined. Numerical simulations and graphical representations are employed to illustrate the implications of our theoretical conclusions.

## Introduction

Epidemiology focuses on understanding how diseases affect population groups, including their causes and health impacts. One such disease, hepatitis B, results from the hepatitis B virus, leading to liver inflammation [1]. Often described as the “silent killer,” hepatitis B may not present symptoms until it is critically advanced. When symptoms do occur, they typically include fever, fatigue, nausea, vomiting, abdominal pain, and joint pain [1, 2]. The time between exposure to the virus and onset of symptoms, or the incubation period, generally spans from 1.5 to 6 months, with an average of about 4 months [2, 3].

In 1942, an outbreak of hepatitis impacted around 28,585 soldiers following their vaccination against yellow fever with a contaminated vaccine [4]. During the entirety of World War II, estimates suggest there were up to 16 million hepatitis cases. Today, the global incidence of new hepatitis B (HBV) infections reaches approximately 4.5 million annually, with a quarter of these leading to liver damage. In 1947, Mccallum and Bauer introduced the terms Hepatitis-A and Hepatitis-B to distinguish between the infectious (epidemic) and serum-based forms of hepatitis [5]. These classifications were formally recognized by the World Health Organization viral hepatitis scientific group in 1973. A significant breakthrough was made at the National Institutes of Health in the USA when Blumberg et al. identified an antigen in the serum of an Australian individual that also reacted with the serum from a patient who had received multiple blood transfusions. This antigen, later named the Hepatitis-B surface antigen (HBsAg) or Australian antigen (Au antigen), was confirmed in 1968 to be uniquely associated with hepatitis B by researchers including Prince, Okochi, and Murakami [6, 7]. Despite advancements in treatment, the mortality rate for acute hepatitis B still ranges from 0.5% to 1%, with HBV causing approximately 600,000 deaths annually [8–10].

Hepatitis B Virus (HBV) can be transmitted through vertical (from mother to infant) and horizontal (via contact with infected blood or other bodily fluids) pathways [11]. HBV presents in two forms: acute and chronic. Acute HBV infection, typically lasting no more than six months, may or may not display symptoms, yet the virus remains transmissible. In most acute cases, the immune system effectively clears the virus. In contrast, chronic HBV persists beyond six months, with the immune system unable to eliminate the virus, which can lead to significant liver damage over time. The main difference between acute and chronic HBV infections lies in their durations. Acute HBV is transient and usually resolves spontaneously, with treatment focused on relieving symptoms and maintaining proper hydration and nutrition, especially following episodes of vomiting and diarrhea. Hepatitis B immunoglobulin (HBIG) is often used in treating acute infections to help prevent the progression to chronic HBV [12]. Chronic HBV requires continuous treatment with oral antiviral medications such as tenofovir or entecavir, which are endorsed by the World Health Organization for effective virus suppression. Managing conditions like cirrhosis that accompany chronic HBV can decelerate disease progression, decrease liver cancer risks, and enhance long-term survival prospects. According to a 2021 WHO report, only 12% to 25% of those eligible for chronic HBV treatment received it [13]. Treatment for hepatitis B is generally lifelong for those who commence it. For chronic hepatitis B or carrier states, treatment strategies extend beyond antiviral medications to include interferon therapy and, in severe cases, liver transplantation. Effective management of hepatitis B is vital; without proper treatment, the condition can lead to severe health issues and potentially fatal outcomes [14–16]. The goal of treatment is not only to suppress the virus and relieve symptoms but also to prevent the disease from progressing to more severe conditions like cirrhosis or liver cancer. This comprehensive approach is crucial to mitigate the long-term impacts of the disease.

Several renowned researchers and investigators employed mathematical modelling to efficiently conduct dynamic analysis with appropriate control procedures. Mathematical modeling has become more crucial for understanding the complex nature of many infectious diseases and determining the most effective approach for controlling them. The objective of developing mathematical models for HBV is to gain insight into the implications of different factors, such as HBV control strategies, HBV vaccination for control purposes, the dynamics of transmission among vaccinated individuals, and the effects of vaccination on hepatitis. Additionally, these models aim to investigate various mathematical frameworks [10, 14, 17]. They have researched the efficacy of vaccination models to analyze costs thoroughly and effectively. The models offered can control HBV in three ways, as referenced by [18, 19]. There has been a surge in global research interest in optimal control problems in recent years. For instance, the authors of [20] examined the *SIR* model to manage the spread of disease. The authors in [21–23] employed optimal control techniques to investigate strategies for preventing and managing various infections. Mathematical modeling is a powerful method for researching the spread of diseases and population dynamics to understand the complex system. This approach can be employed to test and compare various disease transmission patterns while also considering disease control and prevention interventions. Scientists have employed diverse mathematical models in their studies [24–28] to forecast the behavior of different diseases. Mathematical modelling has gained significance in understanding the dynamics of many infectious diseases and selecting the most effective strategy for disease control. Epidemic models serve as valuable tools for elucidating the mechanisms of disease transmission and assessing the efficacy of disease prevention measures. Researchers have utilized several mathematical models, as shown in the references [29–31], to predict the dynamics of various diseases. The utilization of mathematical models demonstrates the benefits of understanding a phenomenon.

All models in the literature study offer different perspectives on HBV dynamics. However, vaccination and treatment for HBV are explored to conduct a more comprehensive examination of the disease, including asymptomatic carriers. Asymptomatic individuals infected with HBV can transmit the virus and result in human mortality during a period of 30 to 180 days. Based on the literature studies [32–34], we have developed a novel HBV model that integrates the assumptions of asymptomatic carriers, vaccination, and treatment training classes. Our model is substantially more sophisticated than the prior model discussed in [35] since we considered the treatment class and successfully obtained the results.

Clinical contribution:

1. Informed Public Health Interventions: Our proposed model can help identify critical parameters (e.g., transmission rates and effectiveness of control measures) that significantly influence disease spread. Understanding these can guide public health authorities in implementing targeted interventions, such as quarantine measures, social distancing guidelines, and vaccination campaigns [36].
2. Vaccination Strategies: The model can be used to assess the effectiveness of vaccination strategies and their impact on reducing disease transmission within the community. This can inform clinical practices by identifying priority groups for vaccination and optimizing vaccination schedules [37].
3. Resource Allocation: By predicting the number of symptomatic individuals and the demand for medical resources (e.g., hospital beds, ventilators), the model can inform healthcare providers and policymakers about resource allocation to ensure adequate healthcare capacity [38].
4. Understanding Disease Dynamics: The model helps understand the dynamics of disease spread, including asymptomatic transmission and the role of different population segments. This can inform clinical guidelines and public health policies to control the spread of the disease [39].

The model is presented in terms of equilibrium points, basic reproduction numbers, existence, uniqueness, and numerical simulation. Section 2 relates to formulating and proving the fundamental results for our proposed model. Section 3 discusses the HBV model analysis, while Section 4 gives a numerical approach to investigating the influence of different parameters on the dynamics of HBV transmission. Finally, our study effort concludes in the last section.

## Model formulation

The field of modeling the dynamics of infectious diseases has shown significant growth in recent years. These efforts help in the prevention of epidemics and the control of diseases. Here in this paper the entire population *N*(*t*) is divided into eight classes to comprehend HBV infection in asymptomatic carriers, vaccination, and treatment classes. These classes include Susceptible S(t), latent L(t), acute infection with symptoms *I*_*a*_(*t*), and asymptomatic infection without visible symptoms. The variables *I*_*ac*_(*t*), *I*_*c*_(*t*), *T*(*t*), *V*(*t*), and *R*(*t*) represent the carriers, treated persons, treatment, vaccination, and recovered individuals, respectively.

$$\begin{array}{c} N\left(t\right)=S\left(t\right)+L\left(t\right)+I_{a}\left(t\right)+I_{ac}\left(t\right)+I_{c}\left(t\right)+T\left(t\right)+V\left(t\right)+R\left(t\right). \end{array}$$

The susceptible population’s *S* change over time is determined by:

$$\begin{array}{c} \frac{dS}{dt}=\Pi -\beta \left(I_{a}+\tau_{1}I_{ac}+\tau_{2}I_{c}\right)S-\nu S+\omega V-\gamma S \end{array}$$

In this case, the rate at which new susceptible individuals are added to the population (e.g., through birth) is Π. *β* is the effective contact rate which leads to new infections. *I*_*a*_, *I*_*ac*_, *I*_*c*_ is the number of asymptomatic infectious, acutely symptomatic infectious, and chronic infectious individuals, respectively. The vaccination rate for susceptible individuals is denoted by *ν*, where *ω* is the rate at which vaccinated individuals return to the susceptible class due to waning immunity. The rate of natural death of susceptible individuals is representing by *γS*.

The rate of change of the latent population over time is determined by:

$$\begin{array}{c} \frac{dL}{dt}=\beta \left(I_{a}+\tau_{1}I_{ac}+\tau_{2}I_{c}\right)S-\left(\gamma +\delta \right)L. \end{array}$$

The rate at which latent individuals become infectious without symptoms is representing by *δ*, where *γL* represent the rate of natural death of latent individuals.

The rate of change of the asymptomatic infectious population over time is determined by:

$$\begin{array}{c} \frac{dI_{a}}{dt}=\delta \kappa L-\left(\gamma +\gamma_{1}+\delta_{4}+\delta_{1}\right)I_{a}. \end{array}$$

completing the incubation period by the individuals joining the acute class a rate *δ*_*κ*_, where *γ*_1_, *δ*_4_ are the rate at which asymptomatic infectious individuals progress to acutely symptomatic infectious state or recover. There *γI*_*a*_ represent the rate of natural death of asymptomatic infectious individuals.

The rate of change of the acutely symptomatic infectious population over time is determined by:

$$\begin{array}{c} \frac{dI_{ac}}{dt}=\delta \left(1-\kappa \right)L-\left(\gamma +\gamma_{2}+\delta_{5}+\delta_{2}\right)I_{ac}. \end{array}$$

Upon completing the incubation period by the individuals joining the asymptomatic carriers class at a rate *δ*(1 − *κ*), where *γ*_2_, *δ*_2_ are the rate at which acutely symptomatic individuals progress to chronic infection or recover. There *γI*_*ac*_ represent the rate of natural death of acutely symptomatic infectious.

The rate of change of the chronic infectious population over time is determined by:

$$\begin{array}{c} \frac{dI_{c}}{dt}=\delta_{4}I_{a}+\delta_{5}I_{ac}-\left(\gamma +\gamma_{3}+\zeta +\delta_{3}\right)I_{c}. \end{array}$$

The rate at which acutely symptomatic infectious individuals become chronic infectious is *δ*_5_, where *ζ* is the rate at which *I*_*c*_ dies due to infection to be vaccinated class. *γ*_3_, *δ*_3_ are the rate of recovery or death of chronic infectious individuals. There *γI*_*c*_ represent the rate of natural death of chronic infectious individuals.

The rate of change of the treatment population over time is determined by:

$$\begin{array}{c} \frac{dT}{dt}=\delta_{1}I_{a}+\delta_{2}I_{ac}+\delta_{3}I_{c}-\left(\gamma +\gamma_{4}\right)T. \end{array}$$

*δ*_1_, *δ*_2_, *δ*_3_ are the rate at which individuals from the infected classes(*I*_*a*_, *I*_*ac*_, *I*_*c*_) start treatment. The rate of recovery or death of treated individuals is representing by *γ*_4_. where *γT* is the rate of natural death of treatment individuals.

The rate of change of the vaccinated population over time is determined by:

$$\begin{array}{c} \frac{dV}{dt}=\nu S+\zeta I_{c}-\left(\omega +\gamma +\gamma_{5}\right)V. \end{array}$$

The rate at which susceptible individuals are vaccinated is *νS*, where *ωV* and *γ*_5_ are the rate at which immunity is lost (vaccinated individuals become susceptible again) or vaccinated individuals, where *γV* is the rate of natural death of vaccinated individuals.

The rate of change of the removed or recovered population over time is determined by:

$$\begin{array}{c} \frac{dR}{dt}=\gamma_{1}I_{a}+\gamma_{2}I_{ac}+\gamma_{3}I_{c}+\gamma_{4}T+\gamma_{5}V-\gamma R. \end{array}$$

**Fig 1:**
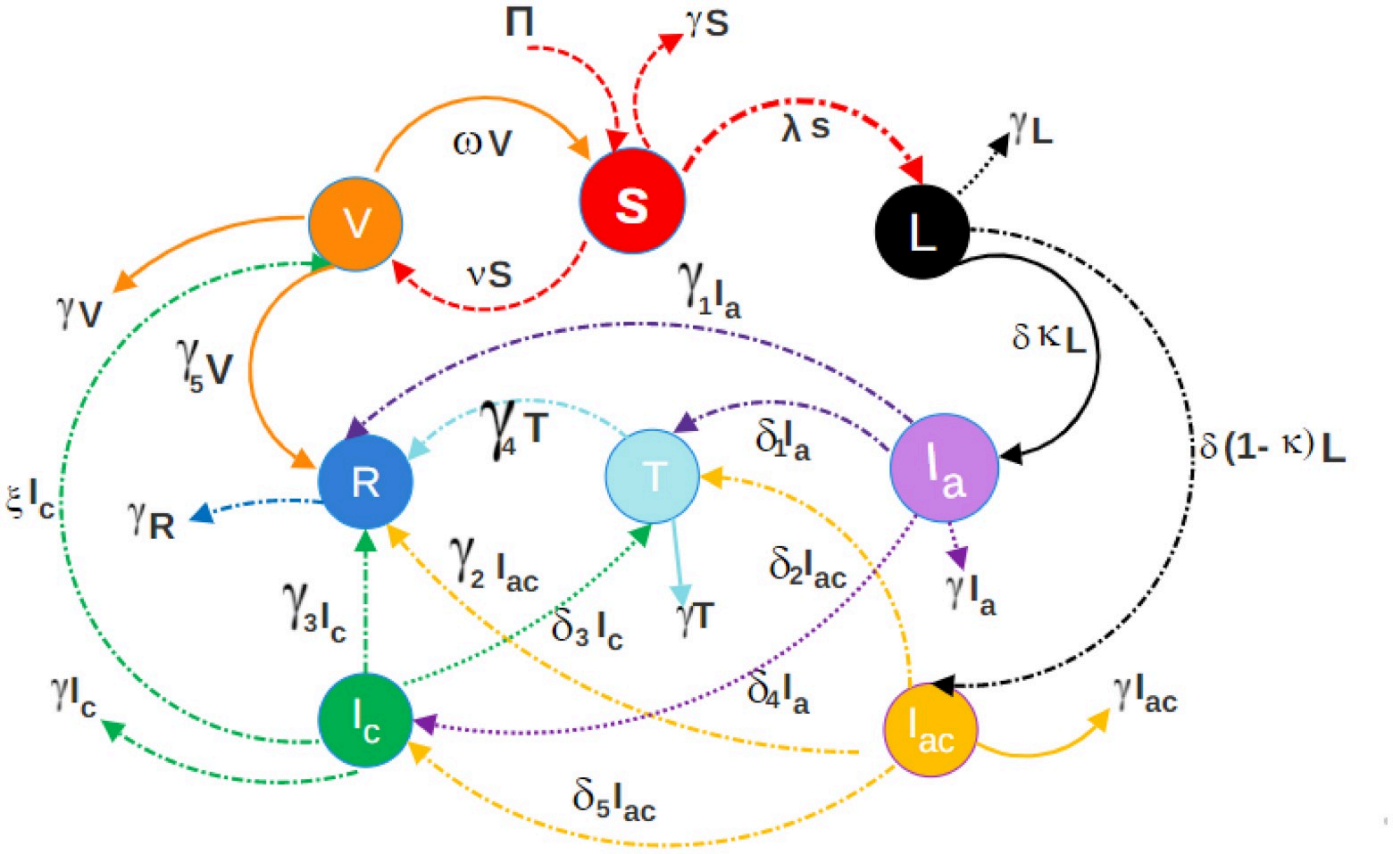
Flowchart illustrating the transmission model of Hepatitis B Virus (HBV).

*γ*_1_, *γ*_2_, *γ*_3_, *γ*_4_, *γ*_5_ are the recovery rates from different infectious states as well as the removal rate for vaccinated individuals, where *γR* is the rate of natural death of recovery individuals. Fig 1 explains the Flowchart Illustrating the Transmission Model of Hepatitis B Virus (HBV).

Here in this paper, we proposed a mathematical model for the dynamics of Hepatitis B Virus (HBV) that includes non-linear differential equations:

$$\begin{array}{c} \left\{ \begin{array}{c} \frac{dS}{dt}=\Pi -\beta \left(I_{a}+\tau_{1}I_{ac}+\tau_{2}I_{c}\right)S-\nu S+\omega V-\gamma S,\\ \frac{dL}{dt}=\beta \left(I_{a}+\tau_{1}I_{ac}+\tau_{2}I_{c}\right)S-\left(\gamma +\delta \right)L,\\ \frac{dI_{a}}{dt}=\delta \kappa L-\left(\gamma +\gamma_{1}+\delta_{4}+\delta_{1}\right)I_{a},\\ \frac{dI_{ac}}{dt}=\delta \left(1-\kappa \right)L-\left(\gamma +\gamma_{2}+\delta_{5}+\delta_{2}\right)I_{ac},\\ \frac{dI_{c}}{dt}=\delta_{4}I_{a}+\delta_{5}I_{ac}-\left(\gamma +\gamma_{3}+\zeta +\delta_{3}\right)I_{c},\\ \frac{dT}{dt}=\delta_{1}I_{a}+\delta_{2}I_{ac}+\delta_{3}I_{c}-\left(\gamma +\gamma_{4}\right)T,\\ \frac{dV}{dt}=\nu S+\zeta I_{c}-\left(\omega +\gamma +\gamma_{5}\right)V,\\ \frac{dR}{dt}=\gamma_{1}I_{a}+\gamma_{2}I_{ac}+\gamma_{3}I_{c}+\gamma_{4}T+\gamma_{5}V-\gamma R. \end{array}\right. \end{array}$$ (1)

With

$$\begin{array}{c} S\left(0\right)=S_{0},\;\;L\left(0\right)=L_{0},\;\;I_{a}\left(0\right)=I_{a0},\;\;I_{ac}\left(0\right)=I_{ac0},\;\;I_{c}\left(0\right)=I_{c0},\;\;T\left(0\right)=T_{0},\;\;V\left(0\right)=V_{0},\;\;R\left(0\right)=R_{0}. \end{array}$$ (2)

The proposed model is:

$$\left\{\begin{array}{l} \frac{dS}{dt}=\Pi -\lambda S(t)-w_{0}S(t)+\omega V(t),\\ \frac{dL}{dt}=\lambda S(t)-w_{1}L(t),\\ \frac{dI_{a}}{dt}=\delta \kappa L(t)-w_{2}I_{a}(t),\\ \frac{dI_{ac}}{dt}=\delta (1-\kappa )L(t)-w_{3}I_{ac}(t),\\ \frac{dI_{c}}{dt}=\delta_{4}I_{a}(t)+\delta_{5}I_{ac}(t)-w_{4}I_{c}(t),\\ \frac{dT}{dt}=\delta_{1}I_{a}(t)+\delta_{2}I_{ac}(t)+\delta_{3}I_{c}(t)-w_{5}T(t),\\ \frac{dV}{dt}=qS(t)+\zeta I_{c}-w_{6}V(t),\\ \frac{dR}{dt}=\nu_{1}I_{a}(t)+\nu_{2}I_{ac}(t)+\nu_{3}I_{c}(t)+\nu_{4}T(t)+\nu_{5}V(t)-\gamma R(t), \end{array}\right.$$ (3)

where

$$\begin{array}{ccc} \lambda & = & \beta \left(I_{a}+\tau_{1}I_{ac}+\tau_{2}I_{c}\right),\;\;w_{0}=\left(\nu +\gamma \right),\;w_{1}=\left(\gamma +\delta \right),\;w_{2}=\left(\gamma +\gamma_{1}+\delta_{1}+\delta_{4}\right),\\ w_{3} & = & \left(\gamma +\gamma_{2}+\delta_{2}+\delta_{5}\right),w_{4}=\left(\gamma +\gamma_{3}+\zeta +\delta_{3}\right),\;\;w_{5}=\left(\gamma +\gamma_{4}\right),\;\;w_{6}=\left(\omega +\gamma +\gamma_{5}\right). \end{array}$$

### 0.1 Boundedness and positivity of solution

In this part, we establish the boundedness and positivity of the state variables in model (3) and also define the feasible region for these variables.

**Theorem 1** *The solution z*(*t*) *of HBV model* (3) *is bounded*

**Proof** The rates of change of total populations are

$$\begin{array}{c} \frac{dN}{dt}=\Pi -\gamma N, \end{array}$$

with

$$\begin{array}{c} N\left(0\right)=S\left(0\right)+L\left(0\right)+I_{0}\left(t\right)+I_{0}\left(t\right)+I_{0}\left(t\right)+T\left(0\right)+V\left(0\right)+R\left(0\right). \end{array}$$

Which implies that

$$\begin{array}{c} \frac{dN}{dt}+\gamma N\leq \Pi . \end{array}$$

Using the ideas of differential inequality introduced in [40], we can write

$$\begin{array}{c} N\left(t\right)\leq \frac{\Pi }{\mu }(1-e^{-\gamma t})+N\left(0\right)e^{-\gamma t}, \end{array}$$

which implies that

$$\begin{array}{c} N\left(t\right)\leq \frac{\Pi }{\mu },\;\;\forall t\geq 0. \end{array}$$

Hence, it is proved that

$$\begin{array}{c} N\left(t\right)\leq \frac{\Pi }{\mu }. \end{array}$$

Thus, the solution *z*(*t*) is bounded for every *t* ≥ 0.

**Theorem 2** *The solution of system of* Eq (3) *having non-negative initial conditions* (2) *is positive* ∀*t* ≥ 0.

**Proof** First, suppose that

$$\begin{array}{c} z(0)=0 \end{array}$$

From (3), we can write

$$\begin{array}{c} \frac{dS}{dt}+\left(\lambda +w_{0}\right)S\left(t\right)=\Pi +\omega V\left(t\right) \end{array}$$

Since the solutions of model (3) are bounded, the above equation can be write

$$\begin{array}{c} \frac{dS}{dt}+G_{1}\left(t\right)=G_{2}\left(t\right). \end{array}$$

where

$$\begin{array}{c} G_{1}\left(t\right)=\lambda +\left(\nu +\gamma \right)\;and\;G_{2}\left(t\right)=\Pi +\omega V\left(t\right), \end{array}$$

from (3), we can write

$$\begin{array}{c} \frac{dS}{dt}+G_{1}\left(t\right)=G_{2}\left(t\right). \end{array}$$

hence

$$\begin{array}{c} S\left(t\right)\times \text{exp}(\int_{0}^{t^{*}}G_{1}\left(t\right)dt)-S\left(0\right)=\int_{0}^{t^{*}}G_{2}\left(t\right)\times \text{exp}(\int_{0}^{u}G_{1}\left(u\right)du)dt, \end{array}$$

so,

$$\begin{array}{c} S\left(t\right)=S\left(0\right)\times \text{exp}(-\int_{0}^{t^{*}}G_{1}\left(t\right)dt)+\text{exp}(-\int_{0}^{t^{*}}G_{1}\left(t\right)dt)\times \int_{0}^{t^{*}}G_{2}\left(t\right)\times \text{exp}(\int_{0}^{u}G_{1}\left(u\right)du)dt>0. \end{array}$$

It is demonstrated that *S*(*t*) is greater than zero for values of *t* that are greater than zero. Furthermore, it can be proven that the remaining sub-classes, namely *L*(*t*), *I*_*a*_(*t*), *I*_*ac*_(*t*), *I*_*c*_(*t*), *T*(*t*), *V*(*t*), and *R*(*t*), are also positive, thus concluding the proof. Thus, the feasible region for the proposed model (3) is specified as follows:

$$\begin{array}{c} O\left(t\right)=S,L,I_{a},I_{ac},I_{c},T,V,R\in R_{+}^{8}:0\leq N\leq \frac{\Pi }{\mu }. \end{array}$$

## Dynamics of the model

### Model equilibria

In this study, we will examine the integer-order system of the Hepatitis B virus, specifically focusing on the disease-free equilibrium (DFE). For this we equate the derivatives $S^{\prime }\left(t\right),\;L^{\prime }\left(t\right),\;I_{a}^{\prime }\left(t\right),\;I_{ac}^{\prime }\left(t\right),\;I_{c}^{\prime }\left(t\right),\;T^{\prime }\left(t\right),\;V^{\prime }\left(t\right)$, and *R*′(*t*) to zero in Eq (3) without infection, resulting in

$$\begin{array}{c} E_{0}\left(S^{0},0,0,0,0,0,V^{0},R^{0}\right)=(\frac{\Pi w_{6}}{\left(w_{0}w_{6}-\gamma \omega \right)},0,0,0,0,0,\frac{\Pi \nu }{\left(w_{0}w_{6}-\gamma \omega \right)},\frac{\Pi \gamma_{5}\nu }{\gamma (w_{0}w_{6}-\gamma \omega )}). \end{array}$$

In addition, we will utilise the method outlined in [41] to calculate the basic reproduction number $R_{0}$.

$$\begin{array}{c} F=(\begin{array}{cccccc} 0 & \beta S(t) & \beta S\left(t\right)\tau_{1} & \beta S\left(t\right)\tau_{2} & 0 & 0\\ 0 & 0 & 0 & 0 & 0 & 0\\ 0 & 0 & 0 & 0 & 0 & 0\\ 0 & 0 & 0 & 0 & 0 & 0\\ 0 & 0 & 0 & 0 & 0 & 0\\ 0 & 0 & 0 & 0 & 0 & 0 \end{array}),\;\;V=(\begin{array}{cccccc} w_{1} & 0 & 0 & 0 & 0 & 0\\ -\delta \kappa & w_{2} & 0 & 0 & 0 & 0\\ -\delta (1-\kappa ) & 0 & w_{3} & 0 & 0 & 0\\ 0 & -\delta_{4} & -\delta_{5} & w_{4} & 0 & 0\\ 0 & -\delta_{1} & -\delta_{2} & -\delta_{3} & w_{5} & 0\\ 0 & 0 & 0 & -\zeta & 0 & w_{6} \end{array}), \end{array}$$

Taking the spectral radius of (**FV**^−1^) which is the basic reproduction number $R_{0}$ at DFE is obtained as:

$$\begin{array}{ccc} R_{0} & = & \frac{\Pi \beta \delta w_{6}\left(\kappa w_{3}\left(w_{4}+\delta_{4}\tau_{2}\right)+w_{2}\left(1-\kappa \right)\left(w_{4}\tau_{1}+\delta_{5}\tau_{2}\right)\right)}{w_{1}w_{2}w_{3}w_{4}\left(\left(\gamma_{5}w_{0}+\gamma \left(\gamma +\nu +\omega \right)\right)\right)}. \end{array}$$ (4)

$R_{1}=\frac{\Pi \beta \delta \kappa w_{6}}{w_{1}w_{2}\left(w_{0}w_{6}-\nu \omega \right)},\;R_{2}=\frac{\Pi \beta \delta \kappa w_{6}\delta_{4}\tau_{2}}{w_{1}w_{2}w_{4}\left(w_{0}w_{6}-\nu \omega \right)},\;R_{3}=\frac{\Pi \beta \delta \left(1-\kappa \right)w_{6}\delta_{5}\tau_{2}}{w_{1}w_{3}w_{4}\left(w_{0}w_{6}-\nu \omega \right)},\;R_{4}=\frac{\Pi \beta \delta \left(1-\kappa \right)w_{6}\tau_{1}}{w_{1}w_{3}\left(w_{0}w_{6}-\nu \omega \right)}.$

### 0.2 Critical vaccination coverage

We examine the Critical Vaccination Coverage rate, which has the potential to eradicate the disease. The value of $R_{0}\left(\nu \right)$ is equal to $R_{E}$. When the vaccination rate in the population is zero, denoted as *ν* = 0, the effective reproduction number decreases to

$$\begin{array}{ccc} R_{0}\left(0\right) & = & \frac{\Pi \beta \delta (\kappa w_{3}\left(w_{4}+\delta_{4}\tau_{2}\right)+w_{2}\left(1-\kappa \right)\left(w_{4}\tau_{1}+\delta_{5}\tau_{2}\right))}{w_{1}w_{2}w_{3}w_{4}\gamma }, \end{array}$$ (5)

where, $R_{0}\left(\nu \right)$ can be written in terms of $R_{0}\left(0\right)$ as

$$\begin{array}{c} R_{0}=R_{0}\left(\nu \right)=\frac{\gamma w_{6}R_{0}\left(0\right)}{\left(\gamma_{5}w_{0}+\gamma \left(\gamma +\nu +\omega \right)\right)}. \end{array}$$

By calculating the partial derivative of $R_{0}\left(\nu \right)$,

$$\begin{array}{c} \frac{\partial R_{0}\left(\nu \right)}{\partial \nu }=-\frac{\gamma w_{6}\left(\gamma +\gamma_{5}\right)}{{\left(\gamma w_{6}+\nu \left(\gamma +\gamma_{5}\right)\right)}^{2}}R_{0}\left(0\right)<0. \end{array}$$

Thus, it follows that $R_{0}\left(\nu \right)$ is less than or equal to $R_{0}\left(0\right)$, and thus, if $R_{0}$ is less than 1, then $R_{0}\left(\nu \right)$ is also less than 1. However, the converse is not necessarily true. If $R_{0}>1$, it indicates that the disease cannot be completely eliminated even with high vaccine coverage, especially when the vaccine efficacy *ν* is poor and $R_{0}$ is significantly higher than one.

### Stability analysis

Now we analyze the stability analysis of the equilibrium point $E_{0}$ of the proposed system (3). Now we examine the stability behavior of the equilibrium $E_{0}$ of the system of Eq (3).

**Theorem 3** *The greatest common divisor of any two non-negative integers q*_1_ *and q*_2_ *is equal to 1. Let us consider the assumption that ς is equal to the ratio* $\frac{q_{1}}{q_{2}}$ , *and define M as q*_2_. *Under this assumption, the DFE (Disease-Free Equilibrium) is locally asymptotically stable when* $|arg\left(\lambda \right)|>\frac{\pi }{2M}$, *where* λ *is the argument of the matrix* $J_{E_{0}}$. *The symbol* λ *represents the possible roots of the characteristic equation*

$$\begin{array}{c} det(Dig\left[\lambda^{M\varsigma }\lambda^{M\varsigma }\lambda^{M\varsigma }\lambda^{M\varsigma }\lambda^{M\varsigma }\lambda^{M\varsigma }\lambda^{M\varsigma }\lambda^{M\varsigma }\right]-J_{D_{0}})=0. \end{array}$$ (6)

**Proof 1** *To achieve the desired result for our proposed model* (3), *we utilize the Jacobian matrix of the system at the DFE*.

$$\begin{array}{c} J_{E_{0}}=(\begin{array}{cccccccc} -w_{0} & 0 & -\beta S(t) & -\beta S\left(t\right)\tau_{1} & -\beta S\left(t\right)\tau_{2} & 0 & \omega & 0\\ \beta & -w_{1} & \beta S(t) & \beta S\left(t\right)\tau_{1} & \beta S\left(t\right)\tau_{2} & 0 & 0 & 0\\ 0 & \delta \kappa & -w_{2} & 0 & 0 & 0 & 0 & 0\\ 0 & \delta (1-\kappa ) & 0 & -w_{3} & 0 & 0 & 0 & 0\\ 0 & 0 & \delta_{4} & \delta_{5} & -w_{4} & 0 & 0 & 0\\ 0 & 0 & \delta_{1} & \delta_{2} & \delta_{3} & -w_{5} & 0 & 0\\ q & 0 & 0 & 0 & \zeta & 0 & -w_{6} & 0\\ 0 & 0 & \gamma_{1} & \gamma_{2} & \gamma_{3} & \gamma_{4} & \gamma_{5} & -\gamma \end{array}). \end{array}$$

Examining determinant Eq (6) indicates

$$\begin{array}{c} \left(\lambda^{q_{1}}+\gamma \right)\left(\lambda^{q_{1}}+w_{5}\right)\left(\lambda^{6q_{1}}+\chi_{1}\lambda^{5q_{1}}+\chi_{2}\lambda^{4q_{1}}+\chi_{3}\lambda^{3q_{1}}+\chi_{4}\lambda^{2q_{1}}+\chi_{5}\lambda^{q_{1}}+\chi_{6}\right)=0. \end{array}$$ (7)

Since −*γ* and −*w*_5_ are clearly negative eigenvalues, the remaining eigenvalues may be obtained by using the last factor of (7). This is the coefficient

$$\begin{array}{cccc} \chi_{1} & = & w_{0}+w_{1}+w_{2}+w_{3}+w_{4}+w_{6},\\ \chi_{2} & = & \left(\left(\nu_{5}w_{0}+\gamma \left(\gamma +\nu +\omega \right)\right)\right)+k_{0}\left(w_{1}+w_{2}+w_{3}+w_{4}\right)+w_{2}\left(w_{3}+w_{4}+w_{6}\right)+w_{6}\left(w_{1}+w_{3}+w_{4}\right)\\ & & +w_{4}\left(w_{1}+w_{3}\right)+w_{1}w_{3}\left(1-R_{4}\right)+w_{1}w_{2}\left(1-R_{1}\right),\\ \chi_{3} & = & \left(w_{1}+w_{2}+w_{3}+w_{4}\right)\left(\left(\nu_{5}w_{0}+\gamma \left(\gamma +\nu +\omega \right)\right)\right)+w_{0}\left(w_{4}\left(w_{1}+w_{3}+w_{6}\right)+w_{2}\left(w_{3}+w_{4}\right)\right)\\ \; & & +w_{4}w_{6}\left(w_{1}+w_{3}\right)+w_{1}w_{2}w_{3}\left(1-\left(R_{1}+R_{4}\right)\right)+w_{1}w_{2}w_{4}\left(1-\left(R_{1}+R_{2}\right)\right)\\ \; & & +w_{1}w_{3}w_{4}\left(1-\left(R_{3}+R_{4}\right)\right)+w_{1}w_{2}\left(w_{0}+w_{6}\right)\left(1-R_{1}\right)+w_{1}w_{3}\left(w_{0}+w_{6}\right)\left(1-R_{4}\right)\\ \; & & +w_{2}\left(w_{6}\left(w_{3}+w_{4}\right)+w_{3}w_{4}\right),\\ \chi_{4} & \; & = & +w_{2}w_{3}w_{4}\left(w_{0}+w_{6}\right)+\left(\left(\nu_{5}w_{0}+\gamma \left(\gamma +\nu +\omega \right)\right)\right)\left(w_{4}\left(w_{1}+w_{2}+w_{3}\right)+w_{2}w_{3}\right)+w_{1}w_{2}w_{3}w_{4}\left(1-R_{0}\right)\\ \; & & +w_{1}w_{2}w_{4}\left(w_{0}+w_{6}\right)\left(1-\left(R_{1}+R_{2}\right)\right)+w_{1}w_{2}\left(\left(\nu_{5}w_{0}+\gamma \left(\gamma +\nu +\omega \right)\right)\right)\left(1-R_{1}\right)\\ \; & & +w_{1}w_{3}w_{4}\left(w_{0}+w_{6}\right)\left(1-\left(R_{3}+R_{4}\right)\right)+w_{0}w_{1}w_{3}w_{4}\left(\left(\nu_{5}w_{0}+\gamma \left(\gamma +\nu +\omega \right)\right)\right)\left(1-R_{4}\right)\\ \; & & +w_{1}w_{2}w_{3}\left(w_{0}+w_{6}\right)\left(1-\left(R_{1}+R_{4}\right)\right),\\ \chi_{5} & = & w_{2}w_{3}w_{4}\left(\left(\nu_{5}w_{0}+\gamma \left(\gamma +\nu +\omega \right)\right)\right)+w_{1}w_{2}w_{3}\left(\left(\nu_{5}w_{0}+\gamma \left(\gamma +\nu +\omega \right)\right)\right)\left(1-\left(R_{1}+R_{4}\right)\right)\\ \; & & +w_{1}w_{2}w_{4}\left(\left(\nu_{5}w_{0}+\gamma \left(\gamma +\nu +\omega \right)\right)\right)\left(1-\left(R_{1}+R_{2}\right)\right)+w_{1}w_{2}w_{3}w_{4}\left(w_{0}+w_{6}\right)\left(1-R_{0}\right)\\ \; & & +w_{1}w_{3}w_{4}\left(\left(\nu_{5}w_{0}+\gamma \left(\gamma +\nu +\omega \right)\right)\right)\left(1-\left(R_{3}+R_{4}\right)\right),\\ \chi_{6} & = & w_{1}w_{2}w_{3}w_{4}\left(\left(\nu_{5}w_{0}+\gamma \left(\gamma +\nu +\omega \right)\right)\right)\left(1-R_{0}\right). \end{array}$$

Given $R_{0}<1$, the arguments of the roots of equations $(\lambda^{q_{1}}+\gamma )=0$, it follows that *χ*_*i*_ for *i* =, …, 6 are positive as well as $(\lambda^{q_{1}}+w_{5})=0$ are similar, that is to say,

$$\begin{array}{c} arg\left(\lambda_{z}\right)=\frac{\pi }{m}+z\frac{2\pi }{m}>\frac{\pi }{M}>\frac{\pi }{2M},where\;\;z=0,1,....,\left(m-1\right). \end{array}$$

In the same way, we can determine that the arguments of the following equation: $(\lambda^{6q_{1}}+\chi_{1}\lambda^{5q_{1}}+\chi_{2}\lambda^{4q_{1}}+\chi_{3}\lambda^{3q_{1}}+\chi_{4}\lambda^{2q_{1}}+\chi_{5}\lambda^{q_{1}}+\chi_{6})=0$ When $R_{0}<1$, then are all found to be greater than $\frac{\pi }{2M}$ otherwise, if $R_{0}>1$, then less than $\frac{\pi }{2M}$. Therefore, for $R_{0}<1$, the DFE is LAS.

**Lemma 1** *If* $R_{0}>1$ , *then the DFE of proposed system* (3) *is unstable*.

### Endemic equilibrium (EE)

The (EE) of (3)denoted by $E_{1}$ and is given as follows:

$$(\frac{\Pi w_{6}+\zeta \omega I_{c}^{*}\left(t\right)}{w_{6}\lambda^{*}+\left(\nu_{5}w_{0}+\gamma \left(\gamma +\nu +\omega \right)\right)},\frac{\lambda^{*}S^{*}}{w_{1}},\frac{\delta \kappa \lambda^{*}S^{*}}{w_{1}w_{2}},\frac{\delta \left(1-\kappa \right)\lambda^{*}S^{*}}{w_{1}w_{3}},\frac{\delta_{4}I_{a}^{*}+\delta_{5}I_{ac}^{*}}{w_{4}},\frac{\delta_{1}I_{a}^{*}+\delta_{2}I_{ac}^{*}+\delta_{3}I_{c}^{*}}{w_{5}},\frac{\Pi \nu +\left(\lambda^{*}+w_{0}\right)\zeta I_{c}^{*}\left(t\right)}{w_{6}\lambda^{*}+\left(\gamma_{5}w_{0}+\gamma \left(\gamma +\nu +\omega \right)\right)}).$$

Furthermore, at endemic steady-state,

$$\begin{array}{c} \lambda^{*}=\beta \left(I_{a}^{*}+\tau_{1}I_{ac}^{*}+\tau_{2}I_{c}^{*}\right). \end{array}$$ (8)

we have

$$\begin{array}{c} d_{1}\lambda^{*}+d_{2}=0, \end{array}$$

where

$$\begin{array}{ccc} d_{1} & = & w_{1}w_{2}w_{3}w_{4}w_{6}-\delta \zeta \omega (\left(1-\kappa \right)w_{2}\delta_{5}+\kappa w_{3}\delta_{4}),\\ d_{2} & = & w_{1}w_{2}w_{3}w_{4}\left(\left(\gamma_{5}w_{0}+\gamma \left(\gamma +\nu +\omega \right)\right)\right)\left(1-R_{0}\right). \end{array}$$

A unique EE exists, and the backward bifurcation phenomena cannot occur if $R_{0}>1$.

**Theorem 4** *The Endemic equilibrium* $E_{1}$ *of the proposed model* (3) *is LAS if* $R_{0}>1$

**Proof 2** *The Jacobian matrix for the system* (3) *is given by*

$$\begin{array}{c} J_{E_{1}}=(\begin{array}{cccccccc} -w_{0}-\lambda^{*} & 0 & -\beta S^{*}\left(t\right) & -\beta S^{*}\left(t\right)\tau_{1} & -\beta S^{*}\left(t\right)\tau_{2} & 0 & \omega & 0\\ \lambda^{*} & -w_{1} & \beta S^{*}\left(t\right) & \beta S^{*}\left(t\right)\tau_{1} & \beta S^{*}\left(t\right)\tau_{2} & 0 & 0 & 0\\ 0 & \delta \kappa & -w_{2} & 0 & 0 & 0 & 0 & 0\\ 0 & \delta (1-\kappa ) & 0 & -w_{3} & 0 & 0 & 0 & 0\\ 0 & 0 & \delta_{4} & \delta_{5} & -w_{4} & 0 & 0 & 0\\ 0 & 0 & \delta_{1} & \delta_{2} & \delta_{3} & -w_{5} & 0 & 0\\ q & 0 & 0 & 0 & \zeta & 0 & -w_{6} & 0\\ 0 & 0 & \gamma_{1} & \gamma_{2} & \gamma_{3} & \gamma_{4} & \gamma_{5} & -\gamma \end{array}), \end{array}$$

*where l*_1_ = βS*(*t*), *l*_2_ = βS*(*t*)*τ*_1_, *l*_3_ = βS*(*t*)*τ*_2_. *The characteristic equation for* $J_{E_{1}}$ *is given by*

$$\begin{array}{c} \left(\lambda +\gamma \right)\left(\lambda +w_{5}\right)\left(\lambda^{6}+b_{1}\lambda^{5}+b_{2}\lambda^{4}+b_{3}\lambda^{3}+b_{4}\lambda^{2}+b_{5}\lambda^{q}+b_{6}\right)=0. \end{array}$$ (9)

*where*

$$\begin{array}{c} \left\{ \begin{array}{c} b_{1}=\lambda^{*}+w_{0}+w_{1}+w_{2}+w_{3}+w_{4}+w_{6},\\ b_{2}=w_{3}w_{4}+\lambda^{*}\left(w_{1}+w_{2}+w_{3}+w_{4}+w_{6}\right)+\left(w_{0}+w_{6}\right)\left(w_{1}+w_{2}+w_{3}+w_{4}\right)\\ +w_{1}\left(w_{2}+w_{3}+w_{4}\right)+w_{2}\left(w_{3}+w_{4}\right)-\delta \left(\kappa l_{2}+\left(1-\kappa \right)l_{3}\right)+\left(w_{0}w_{6}-q\omega \right),\\ b_{3}=w_{3}w_{4}\left(\lambda^{*}+w_{0}+w_{1}+w_{2}\right)+w_{6}\left(\lambda^{*}\left(w_{1}+w_{2}+w_{3}+w_{4}\right)+w_{1}\left(w_{2}+w_{3}+w_{4}\right)+w_{4}\left(w_{2}+w_{3}\right)+w_{2}w_{3}\right)\\ -\delta (\kappa (l_{2}\left(w_{0}+w_{2}+w_{3}+w_{4}+w_{6}\right)+l_{4}\delta_{4})+\left(1-\kappa \right)(l_{3}\left(w_{0}+w_{2}+w_{4}+w_{6}\right)+l_{4}\delta_{5}))\\ +\left(w_{0}w_{6}-q\omega \right)\left(w_{1}+w_{2}+w_{3}+w_{4}\right),\\ b_{4}=\lambda^{*}\left(w_{1}\left(w_{2}\left(w_{3}+w_{4}+w_{6}\right)+w_{6}\left(w_{3}+w_{4}\right)+w_{3}w_{4}\right)+w_{2}w_{6}\left(w_{3}+w_{4}\right)+w_{2}w_{3}w_{4}+w_{3}w_{4}w_{6}\right)\\ +w_{1}w_{2}w_{3}w_{4}-\delta \kappa l_{2}\left(\left(w_{0}w_{6}-q\omega \right)+w_{0}\left(w_{3}+w_{4}\right)+w_{3}\left(w_{4}+w_{6}\right)+w_{4}w_{6}\right)\\ +\left(w_{0}w_{6}-q\omega \right)\left(w_{1}\left(w_{2}+w_{3}+w_{4}\right)+w_{2}\left(w_{3}+w_{4}\right)+w_{3}w_{4}\right)-\delta l_{4}(\left(1-\kappa \right)\delta_{5}\left(w_{0}+w_{2}+w_{6}\right)\\ +\kappa \delta_{4}\left(w_{0}+w_{3}+w_{6}\right))-\delta l_{3}\left(1-\kappa \right)(\left(w_{0}w_{6}-q\omega \right)\\ +w_{2}\left(w_{0}+w_{6}\right)+w_{4}\left(w_{0}+w_{2}+w_{6}\right))+w_{0}\left(w_{1}w_{4}\left(w_{2}+w_{3}\right)+w_{1}w_{2}w_{3}+w_{2}w_{3}w_{4}\right)\\ +w_{6}\left(w_{1}w_{4}\left(w_{2}+w_{3}\right)+w_{1}w_{2}w_{3}+w_{2}w_{3}w_{4}\right),\\ b_{5}=\lambda^{*}w_{6}\left(w_{1}w_{4}\left(w_{2}+w_{3}\right)+w_{2}w_{3}\left(w_{1}+w_{4}\right)\right)-\delta \left(\left(\zeta \lambda^{*}\delta_{5}\omega +\kappa \delta_{4}\right)\left(\zeta \lambda^{*}\omega +l_{4}\left(\left(w_{0}w_{6}-q\omega \right)+w_{3}\left(w_{0}+w_{6}\right)\right)\right)\right)\\ -\delta \left(1-\kappa \right)l_{3}\left(w_{0}w_{6}\left(w_{2}+w_{4}\right)+w_{2}w_{4}\left(w_{0}+w_{6}\right)\right)-\delta \left(1-\kappa \right)\delta_{5}\left(\zeta \lambda^{*}\omega +l_{4}\left(\left(w_{0}w_{6}-q\omega \right)+w_{2}\left(w_{0}+w_{6}\right)\right)\right)\\ +w_{1}w_{2}w_{3}w_{4}\left(w_{0}+w_{6}\right)\left(w_{0}w_{6}-q\omega \right)\left(w_{1}w_{4}\left(w_{2}+w_{3}\right)+w_{2}w_{3}\left(w_{1}+w_{4}\right)\right))\\ +\lambda^{*}w_{1}w_{2}w_{3}w_{4}+\delta \left(1-\kappa \right)l_{3}q\omega \left(w_{2}+w_{4}\right)-\delta l_{2}(\kappa \left(w_{3}+w_{4}\right)\left(w_{0}w_{6}-q\omega \right)\\ +w_{3}w_{4}\left(w_{0}+\kappa w_{6}\right)),\\ b_{6}=\left(w_{3}\left(w_{1}w_{2}\delta +\kappa l_{2}\right)-\delta \left(\left(1-\kappa \right)l_{3}w_{2}\right)\right)-\delta \left(\left(1-\kappa \right)\delta_{5}w_{2}+\kappa \delta_{4}w_{3}\right)\left(\zeta \lambda^{*}\omega +l_{4}\left(w_{0}w_{6}-q\omega \right)\right)\\ +\lambda^{*}w_{1}w_{2}w_{3}w_{4}w_{6}+w_{4}\left(w_{0}w_{6}-q\omega \right). \end{array}\right. \end{array}$$

*Two of the eigenvalues of* $J_{E_{1}}$ , −*γ* *and* −*w*_5_, *are clearly negative based on* (9). *Furthermore, if b*_*i*_ > 0 *for i* = 1, 2, …6, *where M*_1_ = *b*_1_., *it is simple to confirm that the above equation given in* (9) *yields the eigenvalues with negative real portions*.

$$\begin{array}{c} M_{2}=(\begin{array}{cc} b_{1} & 1\\ b_{3} & b_{2} \end{array}),\;\;M_{3}=(\begin{array}{ccc} b_{1} & 1 & 0\\ b_{3} & b_{2} & b_{1}\\ 0 & 0 & b_{3} \end{array})M_{4}=(\begin{array}{cccc} b_{1} & 1 & 0 & 0\\ b_{3} & b_{2} & b_{1} & 0\\ 0 & b_{4} & b_{3} & b_{2}\\ 0 & 0 & 0 & b_{4} \end{array}),\\ M_{5}=(\begin{array}{ccccc} b_{1} & 1 & 0 & 0 & 0\\ b_{3} & b_{2} & b_{1} & 1 & 0\\ b_{5} & b_{4} & b_{3} & b_{2} & b_{1}\\ 0 & 0 & b_{5} & b_{4} & b_{3}\\ 0 & 0 & 0 & 0 & b_{5} \end{array}),\;\;M_{6}=(\begin{array}{cccccc} b_{1} & 1 & 0 & 0 & 0 & 0\\ b_{3} & b_{2} & b_{1} & 1 & 0 & 0\\ b_{5} & b_{4} & b_{3} & b_{2} & b_{1} & 1\\ 0 & b_{6} & b_{5} & b_{4} & b_{3} & b_{2}\\ 0 & 0 & 0 & b_{6} & b_{5} & b_{4}\\ 0 & 0 & 0 & 0 & 0 & b_{6} \end{array}). \end{array}$$

*In this case, for i* = 1, 2, …..6, *all b*_*i*_ > 0 *and M*_*i*_ > 0 *(may be readily modified by utilizing computer algebra package Mathematica). Consequently, the LAS of the EE* $E_{1}$ *of the model* (3) *is guaranteed by the Routh-Hurtwiz criteria*.

### Global stability

To demonstrate that the DFE point $E_{0}$ is globally stable, we follow the approach proposed by Castillo-Chavez et al. [41] and rewrite the proposed model in the form

$$\begin{array}{ccc} \frac{dX}{dt} & = & F\left(X,I\right),\\ \frac{dI}{dt} & = & G(X,I),\;\;G(X,0)=0 \end{array}$$

where X=(S,V) and $I=(L,I_{a},I_{ac},I_{c},T)$, with $X\in R_{+}^{2}$ and $I\in R_{+}^{5}$. We exclude the model’s final equation because it is independent of the others. $E_{0}=\left(\bar{X},0\right)=(\frac{\Pi w_{6}}{\left(w_{0}w_{6}-\gamma \omega \right)},0,0,0,0,0,\frac{\Pi \nu }{\left(w_{0}w_{6}-\gamma \omega \right)})$ is the DFE point. To demonstrate the global asymptotical stability (GAS) of DFE points, the following conditions must be met.

$$\begin{array}{ccc} c_{1}:\frac{dX}{dt} & = & F\left(X,0\right)=0,\;\;\bar{X}\;\;is\;\;GAS,\\ c_{2}:G\left(X,I\right) & = & MI-G\left(\bar{X},I\right),\;\;G\left(\bar{X},I\right)\geq 0,\;\;\forall \left(X,I\right)\in O\left(t\right), \end{array}$$

where O(t) is a feasible region and $M=D_{I}G\left(X,0\right)$ is a matrix with non-negative off-diagonal elements.

**Theorem 5** *If the required condition, i.e, c*_1_ *and c*_2_ *are satisfied, the DFE point* $E_{0}=\left(\bar{X},0\right)$ *of model* (3) *is GAS*.

**Proof 3** *Let* X=(S,V) *uninfected classes and* $I=(L,I_{a},I_{ac},I_{c},T)$ *is infected classes. Then we have*

$$\begin{array}{cc} \frac{dX}{dt}=F\left(X,I\right)=(\begin{array}{c} \Pi -\lambda S\left(t\right)-w_{0}S\left(t\right)+\omega V\left(t\right)\\ \nu S\left(t\right)+\zeta I_{c}-w_{6}V\left(t\right) \end{array}) & at\;\;E_{0}=\left(\bar{X},0\right),\\ & F\left(\bar{X},0\right)=(\begin{array}{c} \Pi -w_{0}\bar{S(t)}+\omega \bar{V(t)}\\ \nu \bar{S(t)}-w_{6}\bar{V(t)} \end{array})=0. \end{array}$$

*Since, we have* $\bar{X}=\left(\bar{S},\bar{V}\right)=(\frac{\Pi w_{6}}{\left(w_{0}w_{6}-\gamma \omega \right)},\frac{\Pi \nu }{\left(w_{0}w_{6}-\gamma \omega \right)}).$ *thus* $\bar{X}$ *is GAS*.

$$\begin{array}{c} \frac{d}{dt}(\begin{array}{c} L(t)\\ I_{a}\left(t\right)\\ I_{ac}\left(t\right)\\ I_{c}\left(t\right)\\ T(t) \end{array})=(\begin{array}{ccccc} -w_{1} & \beta \bar{S(t)} & \beta \tau_{1}\bar{S(t)} & \beta \tau_{2}\bar{S(t)} & 0\\ \delta \kappa & -w_{2} & 0 & 0 & 0\\ \delta (1-\kappa ) & 0 & -w_{3} & 0 & 0\\ 0 & \delta_{4} & \delta_{5} & -w_{4} & 0\\ 0 & \delta_{1} & \delta_{2} & \delta_{3} & -w_{5} \end{array})(\begin{array}{c} L(t)\\ I_{a}\left(t\right)\\ I_{ac}\left(t\right)\\ I_{c}\left(t\right)\\ T(t) \end{array})-(\begin{array}{c} \lambda (\bar{S}\left(t\right)-S\left(t\right))\\ 0\\ 0\\ 0\\ 0 \end{array}), \end{array}$$ (10)

$$\begin{array}{c} \frac{dI}{dt}=MI-G\left(\bar{X},I\right), \end{array}$$

*where*

$$\begin{array}{c} M=(\begin{array}{ccccc} -w_{1} & \beta \bar{S(t)} & \beta \bar{S(t)}\tau_{1} & \beta \bar{S(t)}\tau_{2} & 0\\ \delta \kappa & -w_{2} & 0 & 0 & 0\\ \delta (1-\kappa ) & 0 & -w_{3} & 0 & 0\\ 0 & \delta_{4} & \delta_{5} & -w_{4} & 0\\ 0 & \delta_{1} & \delta_{2} & \delta_{3} & -w_{5} \end{array}),\;\;I=(\begin{array}{c} L(t)\\ I_{a}\left(t\right)\\ I_{ac}\left(t\right)\\ I_{c}\left(t\right)\\ T(t) \end{array}), \end{array}$$ (11)

$$\begin{array}{c} G\left(\bar{X},I\right)=(\begin{array}{c} \lambda (\bar{S}\left(t\right)-S\left(t\right))\\ 0\\ 0\\ 0\\ 0 \end{array}). \end{array}$$ (12)

*Since* $S\left(t\right)\leq \bar{S(t)}$ , *we have* $G(\bar{X},I)\geq 0$. *The matrix* M’*s off-diagonal entries are positive. Therefore*, M *is an M-matrix. Since our conditions c*_1_ *and c*_2_ *are met, we may conclude that* $E_{0}$ *is GAS*.

### Global stability of endemic equilibrium

The proposed model (3) at steady state yielded the following results for global stability:

$$\begin{array}{c} \left\{ \begin{array}{ccc} \Pi & = & \lambda^{**}S^{**}+w_{0}S^{**}-\omega V^{**},\\ w_{1}L^{**} & = & \lambda^{**}S^{**},\\ w_{2}I_{a}^{**} & = & \delta \kappa L^{**},\\ w_{3}I_{ac}^{**} & = & \delta \left(1-\kappa \right)L^{**},\\ w_{4}I_{c}^{**} & = & \delta_{4}I_{a}^{**}+\delta_{5}I_{ac}^{**},\\ w_{5}T^{**} & = & \delta_{1}I_{a}^{**}+\delta_{2}I_{ac}^{**}+\delta_{3}I_{c}^{**},\\ w_{6}V^{**} & = & \nu S^{**}+\zeta I_{c}^{**}. \end{array}\right. \end{array}$$ (13)

$$\begin{array}{c} \lambda^{*}=\beta_{1}\left(I_{a}^{**}+\tau_{1}I_{ac}^{**}+\tau_{2}I_{c}^{**}\right). \end{array}$$

**Theorem 6** *The proposed model* (3) *at unique endemic equilibrium* $E_{1}^{*}=\left(S^{*},L^{*},I_{a}^{*},I_{ac}^{*},I_{c}^{*},T^{*},V^{*}\right)$ *is GAS in* Ω, *if* $R_{0}>1$.

**Proof 4** *Consider the following non-linear Lyapunov function for the model* (3), *considering the derivative and associated unique endemic equilibrium* $E_{1}^{*}$ exist by having $R_{0}>1$.

$$\begin{array}{ccc} L(t) & = & \int_{S^{**}}^{S}\left(1-\frac{S^{**}}{x}\right)dx+\int_{L^{**}}^{L}\left(1-\frac{L^{**}}{x}\right)dx+(\frac{w_{1}}{\delta \kappa })\int_{I_{a}^{**}}^{I_{a}}\left(1-\frac{I_{a}^{**}}{x}\right)dx\\ & + & (\frac{w_{2}}{\delta (1-\kappa )})\int_{I_{ac}^{**}}^{I_{ac}}\left(1-\frac{I_{ac}^{**}}{x}\right)dx+\int_{I_{c}^{**}}^{I_{c}}\left(1-\frac{I_{c}^{**}}{x}\right)dx+\int_{T}^{T}\left(1-\frac{T^{**}}{x}\right)dx+\int_{V^{**}}^{V}\left(1-\frac{V^{**}}{x}\right)dx. \end{array}$$

*Derivative of the non-linear Lyapunov function* L(t) *along the solutions of proposed system* (3) *is*

$$\begin{array}{cc} L^{\prime }\left(t\right) & =(1-\frac{S^{**}}{S})S+(1-\frac{L^{**}}{L})L+(1-\frac{I_{c}^{**}}{I_{c}})I_{c}+(\frac{w_{2}}{\delta (1-\kappa )})(1-\frac{I_{ac}^{**}}{I_{ac}})I_{ac}\\ \; & +(\frac{w_{1}}{\delta \kappa })(1-\frac{I_{a}^{**}}{I_{a}})I_{a}+(1-\frac{T^{**}}{T})T+(1-\frac{V^{**}}{V})V+(1-\frac{R^{**}}{R})R. \end{array}$$

*Using the solution of* (13),

$$\begin{array}{ccc} (1-\frac{S^{**}}{S})S & = & (1-\frac{S^{**}}{S})\left[\Pi -\lambda S-w_{0}S+\omega V\right],\\ \; & = & (1-\frac{S^{**}}{S})\left[\lambda^{**}S^{**}+w_{0}S^{**}-\omega V^{**}-\lambda S-w_{0}S+\omega V\right],\\ \; & = & \beta_{1}S^{**}I_{a}^{**}(1-\frac{S^{**}}{S}-\frac{I_{a}}{I_{a}^{**}}(\frac{S}{S^{**}}-1))+\beta_{1}\tau_{1}S^{**}I_{ac}^{**}(1-\frac{S^{**}}{S}-\frac{I_{ac}}{I_{ac}^{**}}(\frac{S}{S^{**}}-1))\\ \; & & +\beta_{1}\tau_{2}S^{**}I_{c}^{**}(1-\frac{S^{**}}{S}-\frac{I_{c}}{I_{c}^{**}}(\frac{S}{S^{**}}-1))+\omega V^{**}(-1+\frac{S^{**}}{S}-\frac{V}{V^{**}}(\frac{S^{**}}{S}-1)\\ \; & & +w_{0}S^{**}(2-\frac{S}{S^{**}}-\frac{S^{**}}{S}). \end{array}$$ (14)

$$\begin{array}{ccc} (1-\frac{L^{**}}{L})L\left(t\right) & = & \left(1-\frac{L^{**}}{L})(\lambda S-w_{1}L\right)\\ \; & = & (1-\frac{L^{**}}{L})(\lambda S-\lambda S^{**}\frac{L}{L^{**}}),\\ \; & = & \beta_{1}S^{**}I_{a}^{**}(1-\frac{L}{L^{**}}-\frac{I_{a}S}{I_{a}^{**}S^{**}}(\frac{L^{**}}{L}-1))\\ \; & & +\beta_{1}\tau_{1}S^{**}I_{ac}^{**}(1-\frac{L}{L^{**}}-\frac{I_{ac}S}{I_{ac}^{**}S^{**}}(\frac{L^{**}}{L}-1))\\ \; & & +\beta_{1}\tau_{2}S^{**}I_{c}^{**}(1-\frac{L}{L^{**}}-\frac{I_{c}S}{I_{c}^{**}S^{**}}(\frac{L^{**}}{L}-1)). \end{array}$$ (15)

$$\begin{array}{ccc} (\frac{w_{1}}{\delta \kappa })(1-\frac{I_{a}^{**}}{I_{a}})I_{a} & = & (\frac{w_{1}}{\delta \kappa })(1-\frac{I_{a}^{**}}{I_{a}})(\delta \kappa L-w_{2}I_{a}),\\ \; & = & (\frac{w_{1}}{\delta \kappa })(1-\frac{I_{a}^{**}}{I_{a}})(\delta \kappa L-\delta \kappa L^{**}\frac{I_{a}}{I_{a}^{**}}),\\ \; & = & \beta_{1}S^{**}I_{a}^{**}(1-\frac{I_{a}}{I_{a}^{**}}-\frac{L}{L^{**}}(\frac{I_{a}^{**}}{I_{a}}-1))\\ \; & & +\beta_{1}\tau_{1}S^{**}I_{ac}^{**}(1-\frac{I_{a}}{I_{a}^{**}}-\frac{L}{L^{**}}(\frac{I_{a}^{**}}{I_{a}}-1))\\ \; & & +\beta_{1}\tau_{2}S^{**}I_{c}^{**}(1-\frac{I_{a}}{I_{a}^{**}}-\frac{L}{L^{**}}(\frac{I_{a}^{**}}{I_{a}}-1)). \end{array}$$ (16)

$$\begin{array}{ccc} (\frac{w_{1}}{\delta (1-\kappa )})(1-\frac{I_{ac}^{**}}{I_{ac}})I_{ac} & = & (\frac{w_{1}}{\delta (1-\kappa )})(1-\frac{I_{ac}^{**}}{I_{ac}})(\delta \left(1-\kappa \right)L-w_{3}I_{ac}),\\ \; & = & (\frac{w_{1}}{\delta (1-\kappa )})(1-\frac{I_{ac}^{**}}{I_{ac}})(\delta \left(1-\kappa \right)L-\delta \left(1-\kappa \right)L^{**}\frac{I_{ac}}{I_{ac}^{**}}),\\ \; & = & \beta_{1}S^{**}I_{a}^{**}(1-\frac{I_{a}}{I_{a}^{**}}-\frac{L}{L^{**}}(\frac{I_{a}^{**}}{I_{a}}-1))\\ \; & & +\beta_{1}\tau_{1}S^{**}I_{ac}^{**}(1-\frac{I_{a}}{I_{a}^{**}}-\frac{L}{L^{**}}(\frac{I_{a}^{**}}{I_{a}}-1))\\ \; & & +\beta_{1}\tau_{2}S^{**}I_{c}^{**}(1-\frac{I_{a}}{I_{a}^{**}}-\frac{L}{L^{**}}(\frac{I_{a}^{**}}{I_{a}}-1)). \end{array}$$ (17)

$$\begin{array}{ccc} (1-\frac{I_{c}^{**}}{I_{c}})I_{c} & = & (1-\frac{I_{c}^{**}}{I_{c}})(\delta_{4}I_{a}+\delta_{5}I_{ac}-w_{4}I_{c}),\\ \; & = & (1-\frac{I_{c}^{**}}{I_{c}})(\delta_{4}I_{a}+\delta_{5}I_{ac}-\frac{\delta_{4}I_{a}^{**}I_{c}}{I_{c}^{**}}-\frac{\delta_{5}I_{ac}^{**}I_{c}}{I_{c}^{**}}),\\ \; & = & \delta_{4}I_{a}^{**}(1-\frac{I_{c}}{I_{c}^{**}}-\frac{I_{a}}{I_{a}^{**}}(\frac{I_{c}^{**}}{I_{c}}-1))+\delta_{5}I_{ac}^{**}(1-\frac{I_{c}}{I_{c}^{**}}-\frac{I_{ac}}{I_{ac}^{**}}(\frac{I_{c}^{**}}{I_{c}}-1)).\;\;\;\;\;\;\;\;\;\;\; \end{array}$$ (18)

$$\begin{array}{ccc} (1-\frac{T^{**}}{T})T & = & (1-\frac{T^{**}}{T})(\delta_{1}I_{a}+\delta_{2}I_{ac}+\delta_{3}I_{c}-w_{5}T),\\ \; & = & (1-\frac{T^{**}}{T})(\delta_{1}I_{a}+\delta_{2}I_{ac}+\delta_{3}I_{c}-\frac{\delta_{1}I_{a}^{**}T}{T^{**}}-\frac{\delta_{2}I_{ac}^{**}T}{T^{**}}-\frac{\delta_{3}I_{c}^{**}T}{T^{**}}),\\ \; & = & \delta_{1}I_{a}^{**}(1-\frac{T}{T^{**}}+\frac{I_{a}}{I_{a}^{**}}(\frac{T}{T^{**}}-1))+\delta_{2}I_{ac}^{**}(1-\frac{T}{T^{**}}+\frac{I_{ac}}{I_{ac}^{**}}(\frac{T}{T^{**}}-1))\\ \; & & +\delta_{3}I_{c}^{**}(1-\frac{T}{T^{**}}+\frac{I_{c}}{I_{c}^{**}}(\frac{T}{T^{**}}-1)).\;\;\;\;\;\;\;\;\;\;\; \end{array}$$ (19)

$$\begin{array}{ccc} (1-\frac{V^{**}}{V})V & = & (1-\frac{V^{**}}{V})(\nu S+\zeta I_{c}-w_{6}V),\\ \; & = & (1-\frac{V^{**}}{V})(\nu S+\zeta I_{c}-\frac{\nu S^{**}V}{V^{**}}-\frac{\zeta I_{c}^{**}V}{V^{**}}),\\ \; & = & \nu S^{**}(1-\frac{V}{V^{**}}-\frac{S}{S^{**}}(\frac{V^{**}}{V}-1))+\zeta I_{c}^{**}(1-\frac{V}{V^{**}}-\frac{I_{c}}{I_{c}^{**}}(\frac{V^{**}}{V}-1)).\;\;\;\;\;\;\;\;\;\;\; \end{array}$$ (20)

*after substituting, we get*

$$\begin{array}{ccc} L^{\prime } & = & \beta_{1}S^{**}I_{a}^{**}(4-\frac{S^{**}}{S}-\frac{I_{ac}}{I_{ac}^{**}}-\frac{L^{**}I_{a}S}{LI_{a}^{**}S^{**}}-\frac{I_{a}^{**}L}{I_{a}L^{**}}-\frac{I_{ac}^{**}L}{I_{ac}L^{**}}+\frac{L}{L^{**}})\\ \; & & +\beta_{1}\tau_{1}S^{**}I_{ac}^{**}(4-\frac{S^{**}}{S}-\frac{I_{a}}{I_{a}^{**}}-\frac{L^{**}I_{ac}S}{LI_{ac}^{**}S^{**}}-\frac{I_{a}^{**}L}{I_{a}L^{**}}-\frac{I_{ac}^{**}L}{I_{ac}L^{**}}+\frac{L}{L^{**}})\\ \; & & +\beta_{1}\tau_{2}S^{**}I_{c}^{**}(4-\frac{S^{**}}{S}-\frac{I_{a}}{I_{a}^{**}}-\frac{I_{ac}}{I_{ac}^{**}}-\frac{L^{**}I_{c}S}{LI_{c}^{**}S^{**}}-\frac{I_{a}^{**}L}{I_{a}L^{**}}-\frac{I_{ac}^{**}L}{I_{ac}L^{**}}+\frac{I_{c}}{I_{c}^{**}}+\frac{L}{L^{**}})\\ \; & & +\omega V^{**}(-1+\frac{S^{**}}{S}-\frac{V}{V^{**}}(\frac{S^{**}}{S}-1)+w_{0}S^{**}(2-\frac{S}{S^{**}}-\frac{S^{**}}{S})\\ \; & & +\delta_{4}I_{a}^{**}(1-\frac{I_{c}}{I_{c}^{**}}-\frac{I_{a}}{I_{a}^{**}}(\frac{I_{c}^{**}}{I_{c}}-1))+\delta_{5}I_{ac}^{**}(1-\frac{I_{c}}{I_{c}^{**}}-\frac{I_{ac}}{I_{ac}^{**}}(\frac{I_{c}^{**}}{I_{c}}-1))\\ \; & & +\delta_{1}I_{a}^{**}(1-\frac{T}{T^{**}}+\frac{I_{a}}{I_{a}^{**}}(\frac{T}{T^{**}}-1))+\delta_{2}I_{ac}^{**}(1-\frac{T}{T^{**}}+\frac{I_{ac}}{I_{ac}^{**}}(\frac{T}{T^{**}}-1))\\ \; & & +\delta_{3}I_{c}^{**}(1-\frac{T}{T^{**}}+\frac{I_{c}}{I_{c}^{**}}(\frac{T}{T^{**}}-1))+\nu S^{**}(1-\frac{V}{V^{**}}-\frac{S}{S^{**}}(\frac{V^{**}}{V}-1))\\ \; & & +\zeta I_{c}^{**}(1-\frac{V}{V^{**}}-\frac{I_{c}}{I_{c}^{**}}(\frac{V^{**}}{V}-1)). \end{array}$$ (21)

*Using the arithmetic geometrical inequality, we get the following interpretation from* Eq (21),

$$\begin{array}{c} \left\{ \begin{array}{c} (2-\frac{S^{**}}{S}-\frac{S}{S^{**}})\leq 0,(\frac{V}{V^{**}}+\frac{S^{**}}{S}-\frac{S^{**}V}{SV^{**}}-1)\leq 0,\\ (1-\frac{I_{c}}{I_{c}^{**}}-\frac{I_{a}}{I_{a}^{**}}(\frac{I_{c}^{**}}{I_{c}}-1))\leq 0,(1-\frac{I_{c}}{I_{c}^{**}}-\frac{I_{ac}}{I_{ac}^{**}}(\frac{I_{c}^{**}}{I_{c}}-1)\leq 0,\\ (1-\frac{V}{V^{**}}-\frac{S}{S^{**}}(\frac{V^{**}}{V}-1))\leq 0,(1-\frac{V}{V^{**}}-\frac{I_{c}}{I_{c}^{**}}(\frac{V^{**}}{V}-1))\leq 0,\\ (1-\frac{T}{T^{**}}+\frac{I_{a}}{I_{a}^{**}}(\frac{T}{T^{**}}-1))\leq 0,(1-\frac{T}{T^{**}}+\frac{I_{ac}}{I_{ac}^{**}}(\frac{T}{T^{**}}-1))\leq 0,\\ (1-\frac{T}{T^{**}}+\frac{I_{c}}{I_{c}^{**}}(\frac{T}{T^{**}}-1))\leq 0,\nu S^{**}(1-\frac{V}{V^{**}}-\frac{S}{S^{**}}(\frac{V^{**}}{V}-1))\leq 0,\\ (4-\frac{S^{**}}{S}-\frac{I_{ac}}{I_{ac}^{**}}-\frac{L^{**}I_{a}S}{LI_{a}^{**}S^{**}}-\frac{I_{a}^{**}L}{I_{a}L^{**}}-\frac{I_{ac}^{**}L}{I_{ac}L^{**}}+\frac{L}{L^{**}})\leq 0,\\ (4-\frac{S^{**}}{S}-\frac{I_{a}}{I_{a}^{**}}-\frac{L^{**}I_{ac}S}{LI_{ac}^{**}S^{**}}-\frac{I_{a}^{**}L}{I_{a}L^{**}}-\frac{I_{ac}^{**}L}{I_{ac}L^{**}}+\frac{L}{L^{**}})\leq 0,\\ (4-\frac{S^{**}}{S}-\frac{I_{a}}{I_{a}^{**}}-\frac{I_{ac}}{I_{ac}^{**}}-\frac{L^{**}I_{c}S}{LI_{c}^{**}S^{**}}-\frac{I_{a}^{**}L}{I_{a}L^{**}}-\frac{I_{ac}^{**}L}{I_{ac}L^{**}}+\frac{I_{c}}{I_{c}^{**}}+\frac{L}{L^{**}})\leq 0. \end{array}\right. \end{array}$$ (22)

Eq (22) *shows that* $L^{\prime }\left(t\right)\leq 0$ *for nonnegative model parameters. If* $R_{0}>1$ , *the LaSalle’s invariance principle* [42] *states that as t* → ∞, $\left(S,L,I_{a},I_{ac},I_{c},T,V,R\right)\to \left(S^{**},L^{**},I_{a}^{**},I_{ac}^{**},I_{c}^{**},T^{**},V^{**},R^{**}\right)$.

## Numerical scheme and discussion

We use non-standard finite difference method (NSFD) to simulate our theoretical results. The concerned method is a powerful numerical tool which has been used in large number to deal mathematical models. The presented scheme for a general class of differential equation is presented as: Let *t*_*k*_ = *kh*, where *h* be the step size, then for the following problem

$$\begin{array}{c} \frac{dx}{dt}=f\left(t,x\left(t\right)\right),t\in \left[0,T\right], \end{array}$$ (23)

the left side can be decomposed as

$$\begin{array}{c} \frac{dx}{dt}=\frac{x_{k+1}-x_{k}}{\psi (h)}, \end{array}$$ (24)

where *ψ*(*h*) [43, 44]

$$\begin{array}{c} \psi \left(h\right)=1-e^{-h}. \end{array}$$ (25)

Using (24) in (23), we get

$$\begin{array}{c} x_{k+1}=x_{k}+\psi \left(h\right)\left[f\left(t_{k},x_{k}\right)\right]. \end{array}$$ (26)

If we express (27) as by taking *h* is very small such that *h*^2^, *h*^3^ and terms with higher orders of *h* are neglected such that

$$\begin{array}{c} \psi (h)\approx h. \end{array}$$ (27)

Then the required numerical scheme (28) can be described as

$$\begin{array}{c} x_{k+1}\approx x_{k}+h\left[f\left(t_{k},x_{k}\right)\right]. \end{array}$$ (28)

Following the mentioned procedure, we can simulate the proposed model by using the following values for the nomenclature.

Using the numerical values of Table 1 and simulate the results for different compartments of the model.

**Table 1:** Parameters values.

Parameters	Values	Ref	Parameters	Values	Ref
∏	0.58	Assumed	*ζ*	0.02	Assumed
*γ*	1/67.7	[35]	*ω*	0.001	[35]
*β*	0.00002	[35]	*γ*_1_	0.001	Assumed
*κ*	0.002	[35]	*γ*_2_	0.001	[35]
*δ*	0.02	[35]	*γ*_3_	0.002	[35]
*τ*_1_	0.02	[35]	*γ*_4_	0.0001	Assumed
*τ*_2_	0.02	[35]	*γ*_5_	0.00002	Assumed
*ν*	0.002	[35]	*δ*_1_	0.002	[35]
*δ*_2_	0.0002	[35]	*δ*_3_	0.0002	Assumed
*δ*_4_	0.0002	[35]	*δ*_5_	0.00003	Assumed

**Fig 2:**
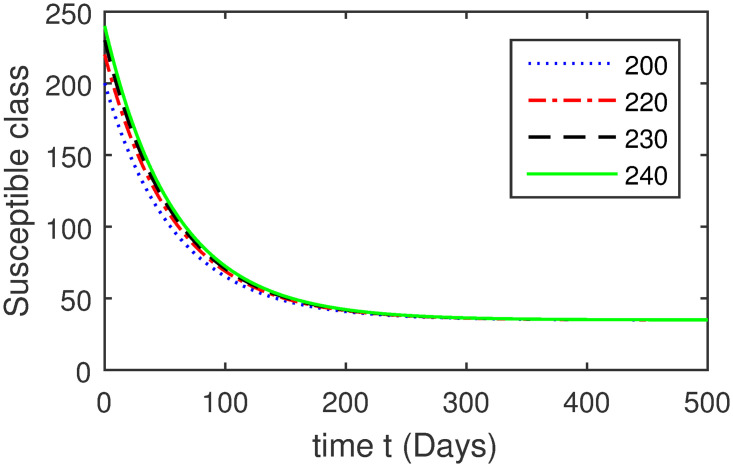
Graphical presentation of *S* class using different values of the parameters.

**Fig 3:**
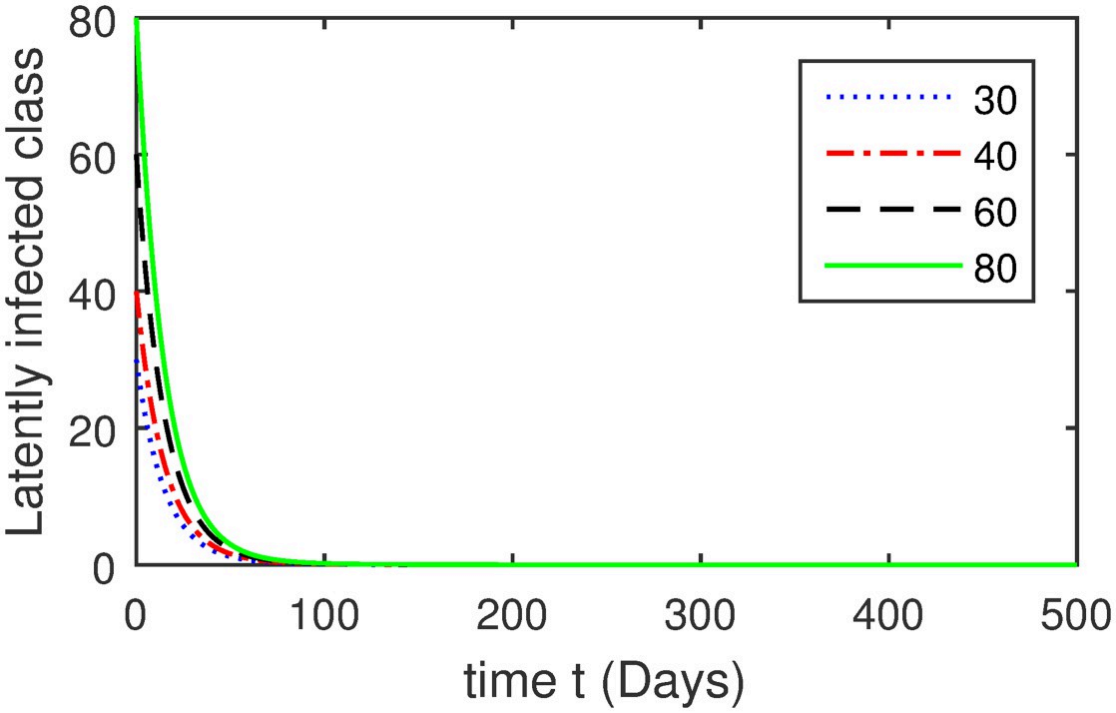
Graphical presentation of *L* class using different values of the parameters.

**Fig 4:**
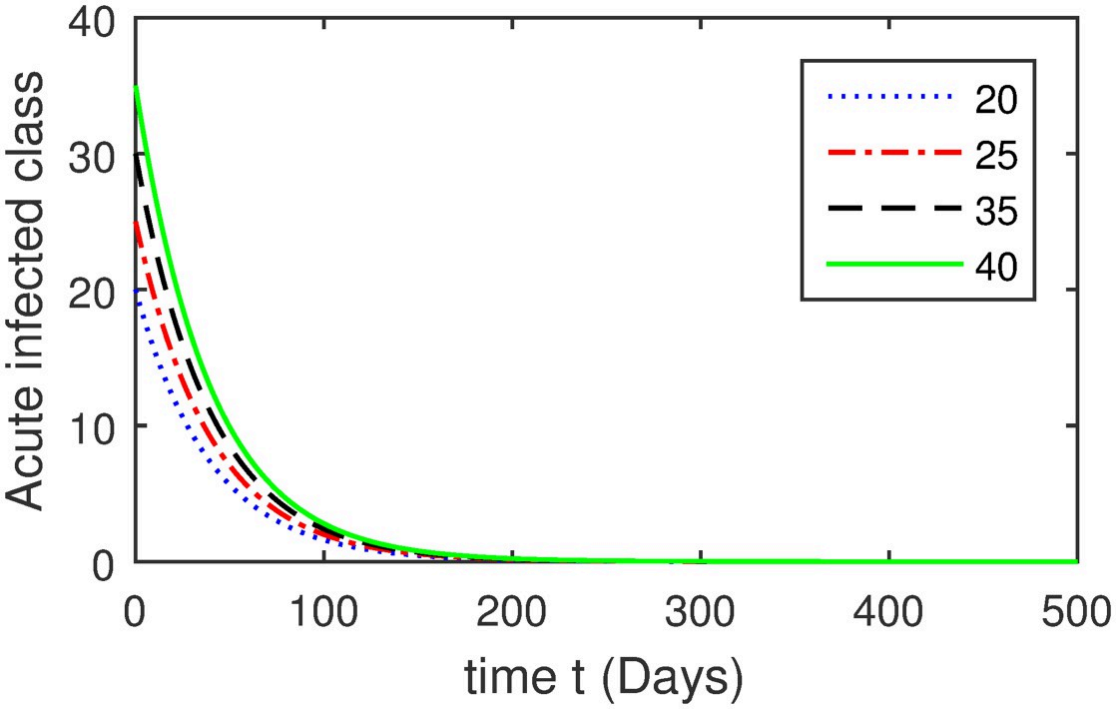
Graphical presentation of *I*_*a*_ class using different values of the parameters.

**Fig 5:**
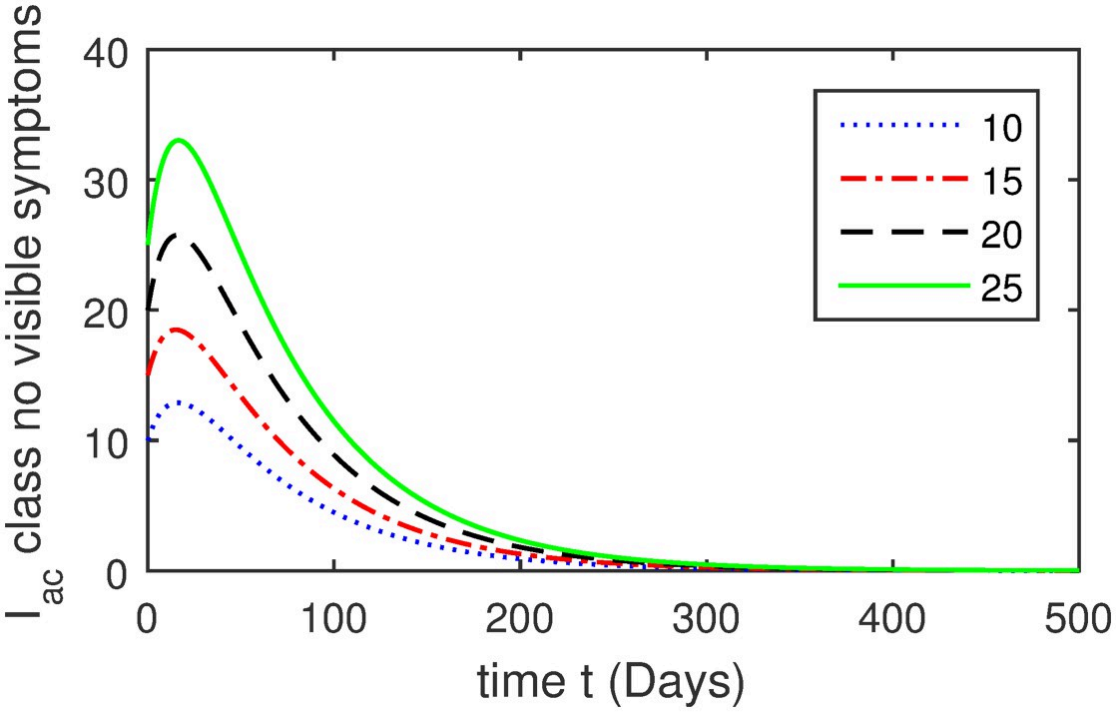
Graphical presentation of *I*_*ac*_ using different values of the parameters.

**Fig 6:**
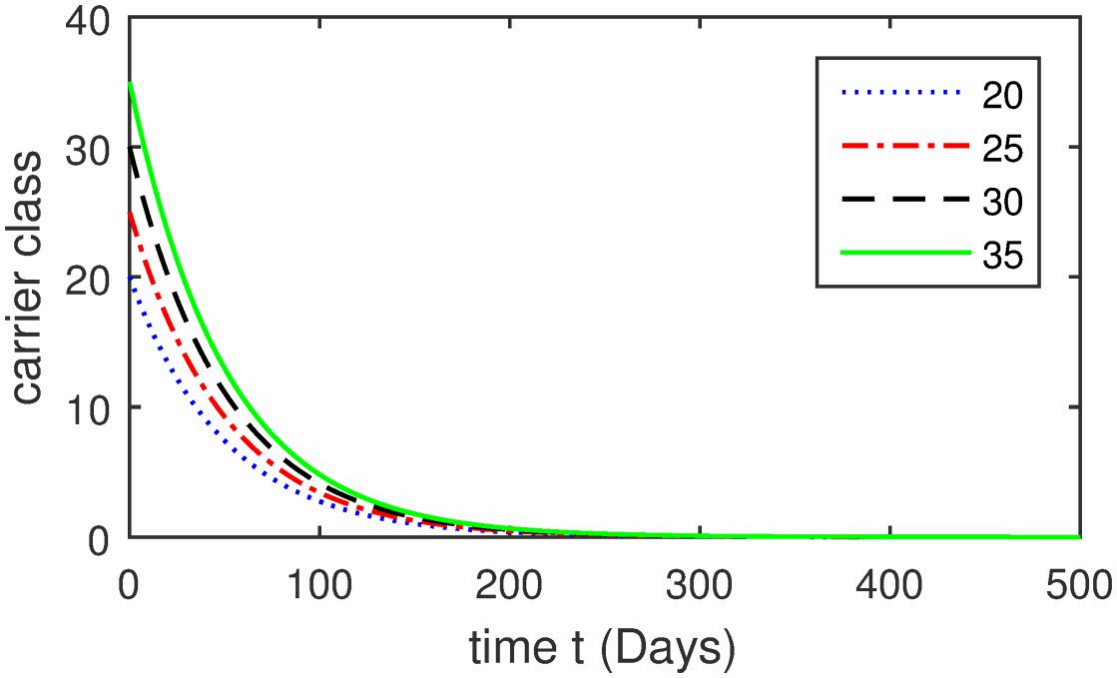
Graphical presentation of *I*_*a*_ using different values of the parameters.

**Fig 7:**
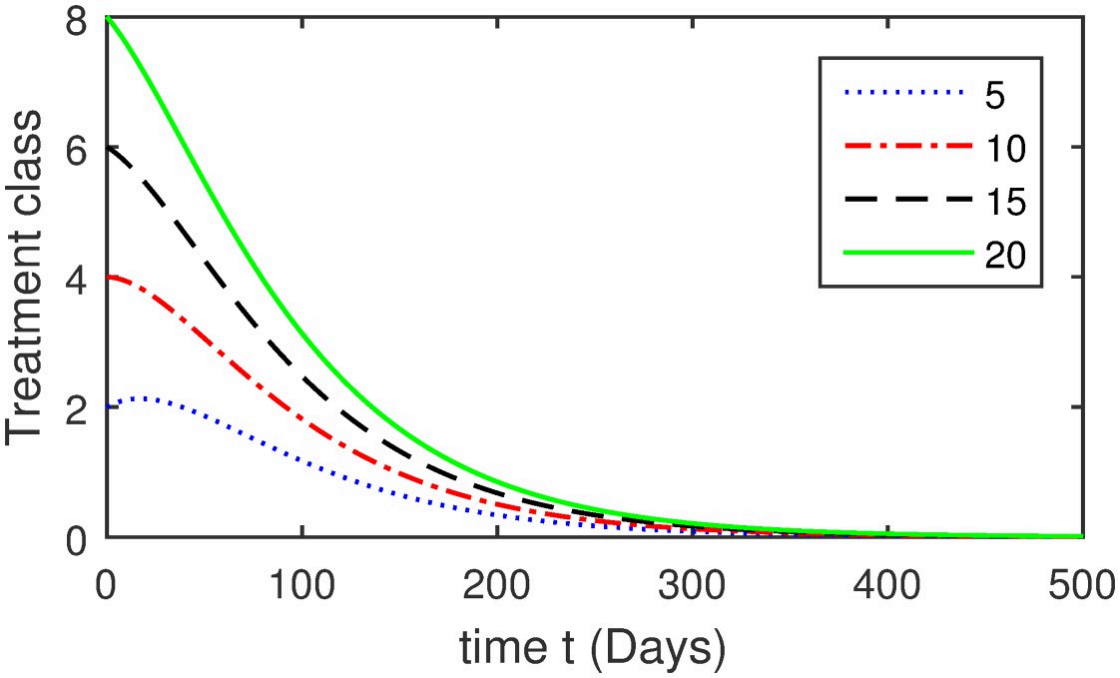
Graphical presentation of *T* using different values of the parameters.

**Fig 8:**
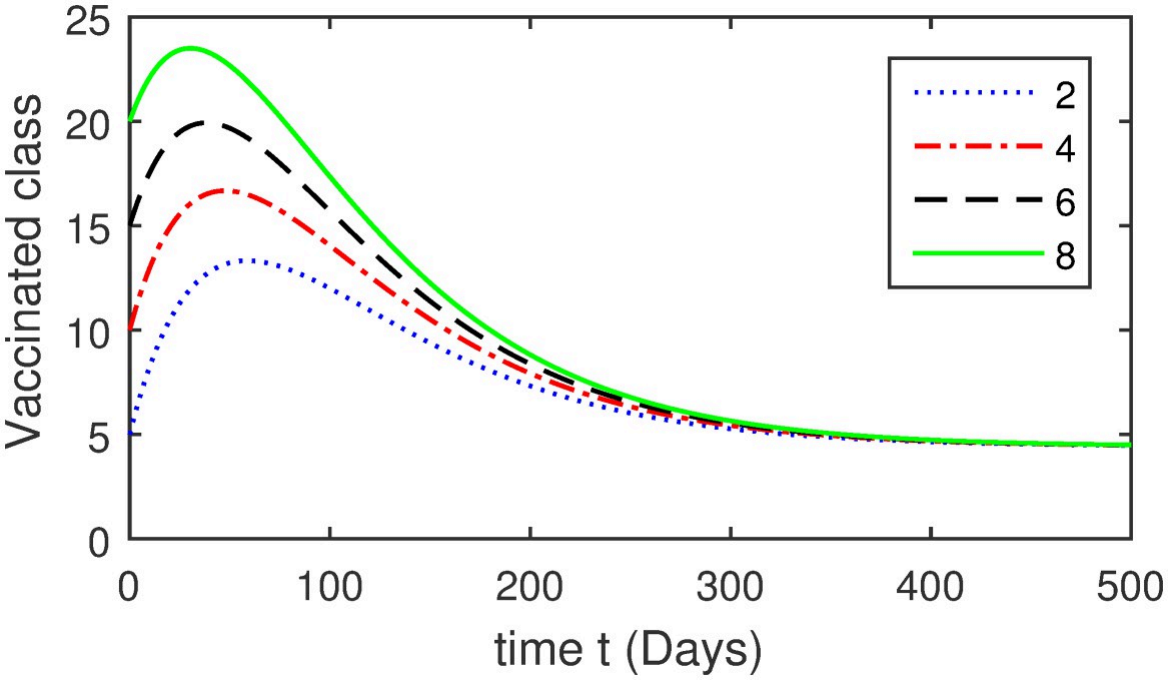
Graphical presentation of *V* using different values of the parameters.

**Fig 9:**
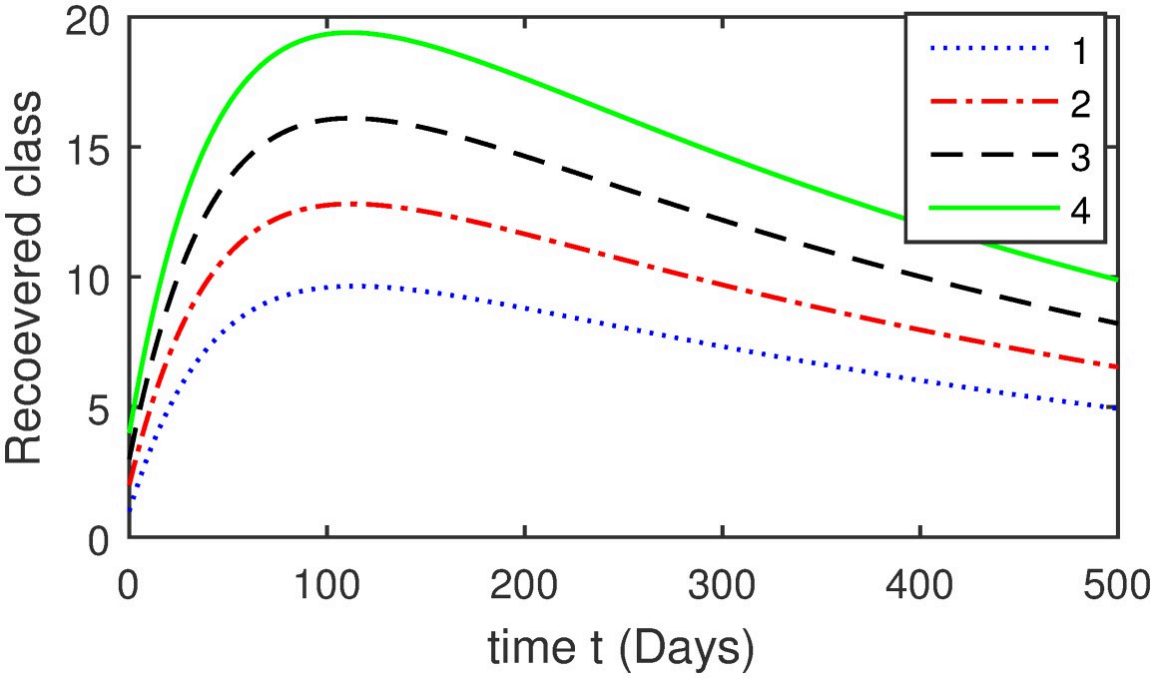
Graphical presentation of *R* using different values of the parameter.

**Fig 10:**
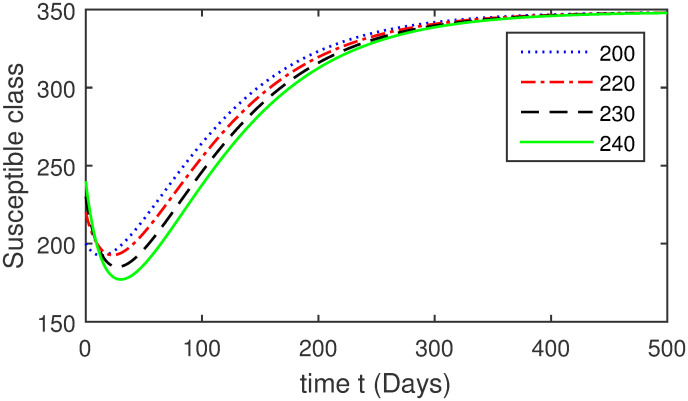
Susceptible class using the numerical scheme when *β* = 0.0009.

**Fig 11:**
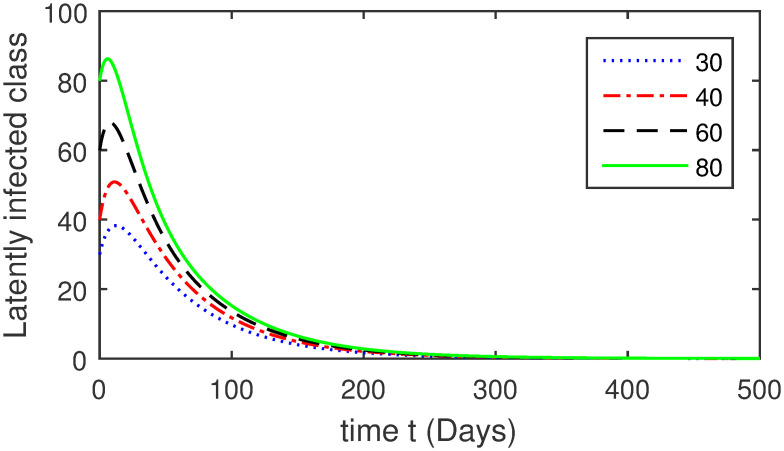
Graphical presentation of latently infected class using the numerical scheme when ∏ = 5.5 and *β* = 0.0009.

**Fig 12:**
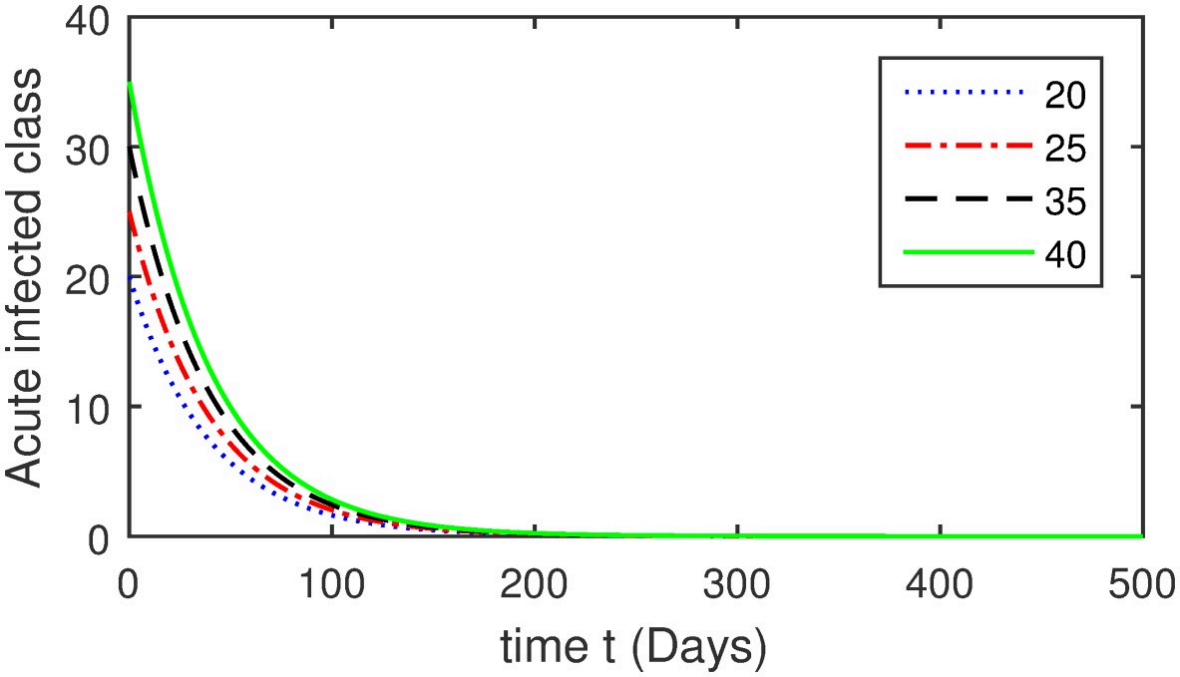
Graphical presentation of acute infected class using the numerical scheme when ∏ = 5.5 and *β* = 0.0009.

**Fig 13:**
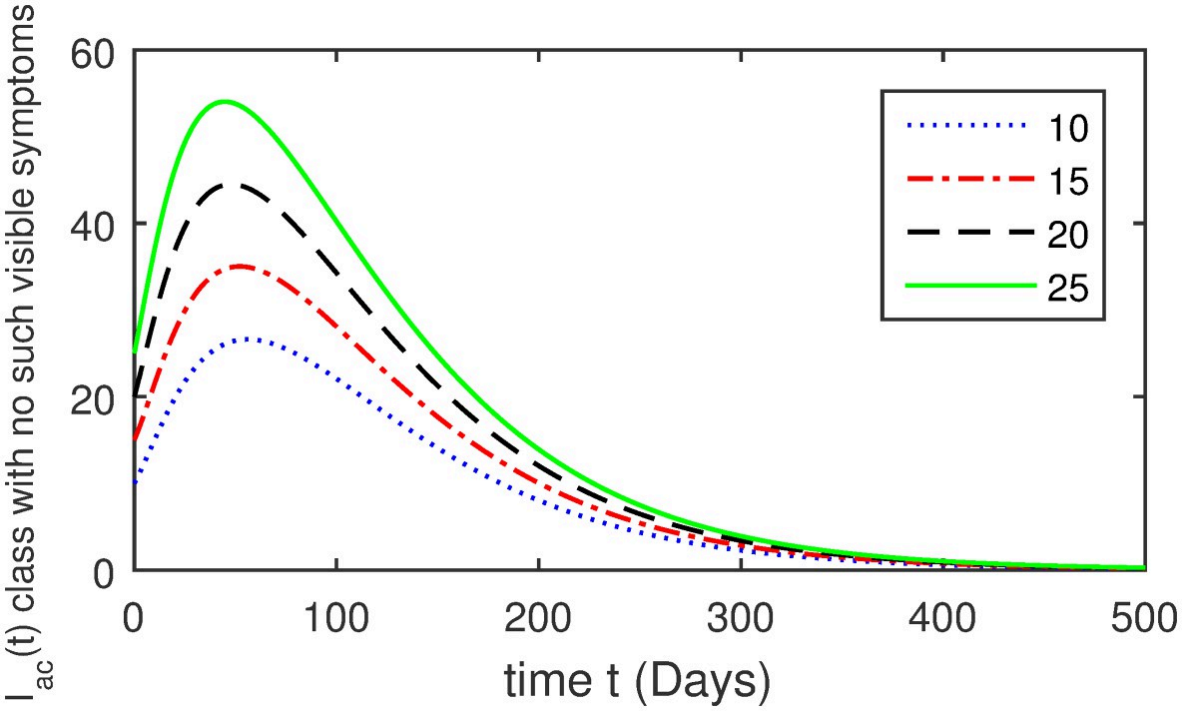
Graphical presentation of asymptotically infected class with no visible symptoms using the numerical scheme when ∏ = 5.5 and *β* = 0.0009.

**Fig 14:**
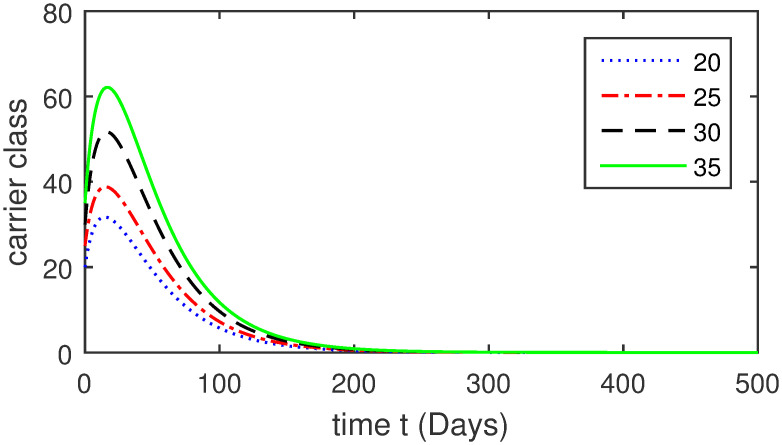
Graphical presentation of carrier-class using the numerical scheme when ∏ = 5.5 and *β* = 0.0009.

**Fig 15:**
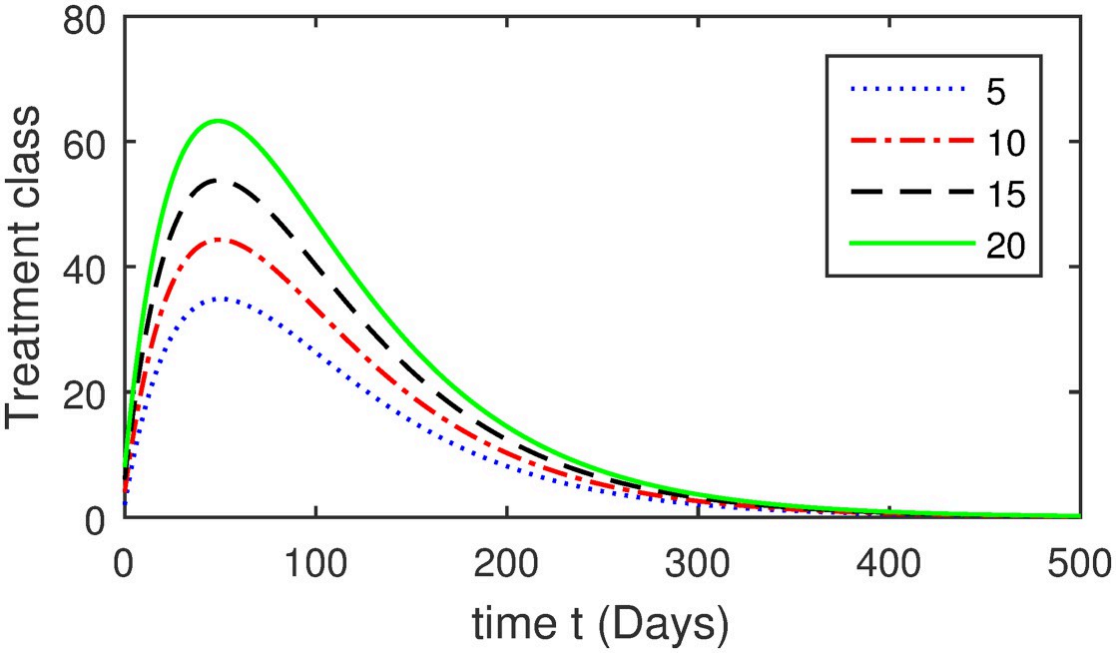
Graphical presentation of treatment class using the numerical scheme when ∏ = 5.5 and *β* = 0.0009.

**Fig 16:**
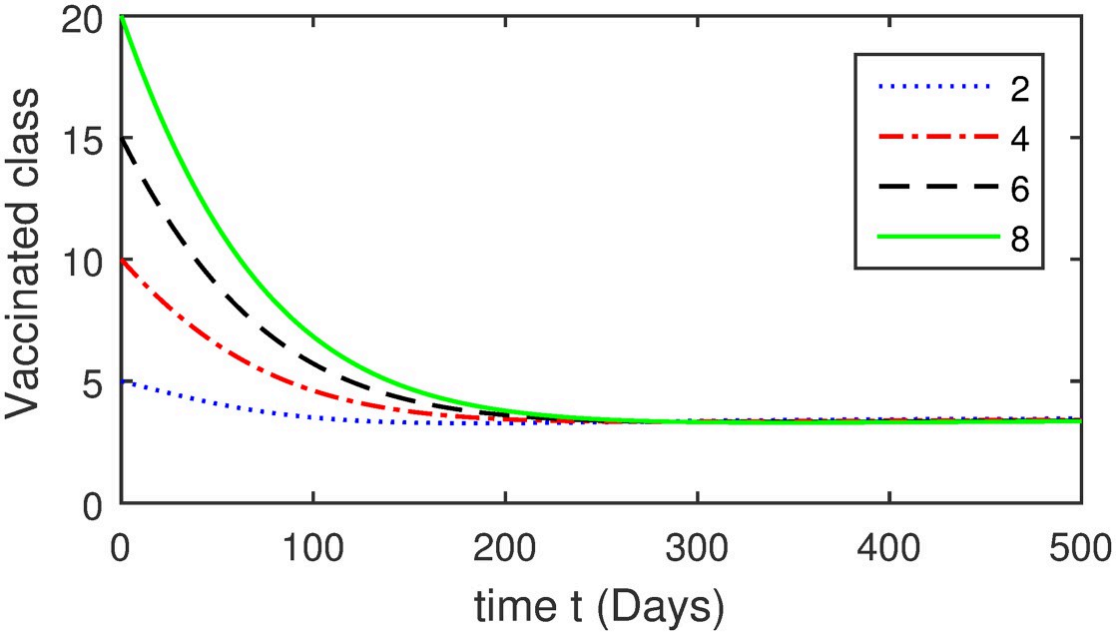
Graphical presentation of vaccinated class using the numerical scheme when ∏ = 5.5 and *β* = 0.0009.

**Fig 17:**
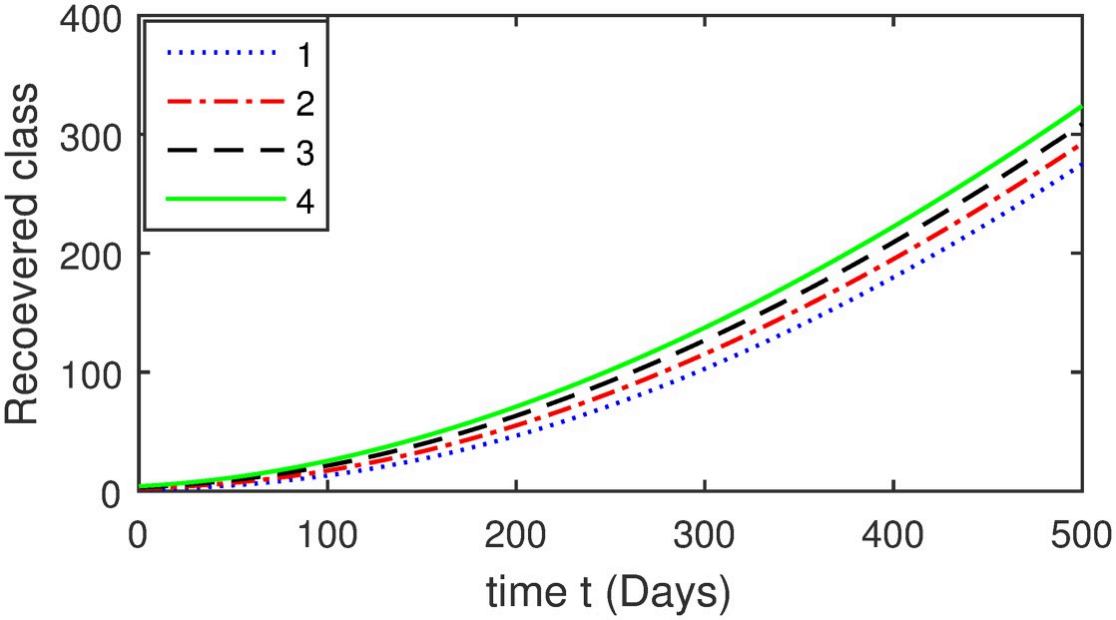
Graphical presentation of recovered class using the numerical scheme when ∏ = 5.5 and *β* = 0.0009.

Here, we provide a view of the model’s (3) numerical simulations. Initial conditions are provided along with the parametric values that are taken into account during the simulation process. *S*(0) = 200, 220, 230, 240, *L*(0) = 30, 40, 60, 80, *I*_*a*_(0) = 20, 25, 30, 35, *I*_*ac*_(0) = 10, 15, 20, 25, *I*_*c*_(0) = 20, 25, 30, 35, *V*(0) = 5, 10, 15, 20, *T*(0) = 2, 4, 6, 8, and *R*(0) = 1, 2, 3, 4. The parametric values used for the sake of simulation are shown in the Figs 2–9 are as follows: ∏ = 0.58, *ζ* = 0.02, *γ* = 1/67.7, *ω* = 0.001, *β* = 0.00002, *γ*_1_ = 0.001, *κ* = 0.002, *γ*_2_ = 0.001, *δ* = 0.02, *γ*_3_ = 0.002, *τ*_1_ = 0.02, *γ*_4_ = 0.0001, *τ*_2_ = 0.02, *γ*_5_ = 0.00002, *ν* = 0.002, *δ*_1_ = 0.002, *δ*_2_ = 0.0002, *δ*_3_ = 0.0002, *δ*_4_ = 0.0002 and *δ*_5_ = 0.00003. For the Figs 10–17 we change the values of the parameter ∏ from 0.58 to 5.5 and *β* from 0.00002 to = 0.0009, and the model yields a better effect on the susceptible population. We have presented various compartments for different initial values in Figs 2–17 respectively. In Figs 2, 3, 10 and 11 the decay in susceptible class as well as in latently infected compartment has shown. The susceptible class graph indicates a decrease in the number of persons at risk of developing Hepatitis B, highlighting the importance of continued public health programs such as immunization and education in reducing those who are susceptible. The latent infection graph explains how the disease develops in those who are infected but do not yet have symptoms. Understanding the duration of opportunity for intervention before the condition becomes more difficult to control is contingent on how this class decreases with time. The acute infected class, asymptotically infected class with no visible symptoms, and carrier class with different initial data have presented graphically their numerical simulations in Figs 4–6, 12–14 respectively. The number of newly infected people rapidly declines in the acute infected class graph, illustrating the efficiency of early intervention strategies. This trend emphasizes the importance of early detection and treatment, demonstrating that treatment and isolation of infected individuals can significantly reduce the spread of Hepatitis B. The results obtained for carrier-class and asymptomatic carriers indicate that the virus is still present in the community, however at a decreased rate. Controlling Hepatitis B spread becomes more challenging when dealing with asymptomatic individuals who may unconsciously spread the infection. Intensive testing and monitoring are crucial for recognizing and tracking cases, limiting the possibility of undetected transmission. Figs 7–9, 15–17 show approximate solutions for various initial values of treatment, vaccinated, and recovered classes. The treatment class graph shows how fewer patients need medical care over time, indicating how effective medications have been in controlling the disease. However, the early spike suggests a pressure on medical resources that could be relieved by immunization, which vaccination and other preventative measures could help to mitigate. The recovered class plot, which shows a positive increase in the number of persons who fully recovered from the infection, contributes to herd immunity. This pattern shows how effective treatment options, together with natural healing, can result in long-term disease control. It’s not a standardized situation, and your numerical simulations have revealed the intricacies that must be considered when developing public health interventions. Our numerical simulations clarified the dynamics of the transmission of HBV and identified key factors for control approaches. In-depth analysis is crucial for creating effective public health policies and interventions.

## Conclusion

Hepatitis, which is defined by liver inflammation, can be caused by a variety of viral and nonviral causes, including drugs, toxins, and immunological responses that target hepatocytes. This study looks at the complex dynamics of Hepatitis B Virus (HBV) infection using a newly developed model that takes into account asymptomatic carriers, vaccination, and treatment. The inclusion of asymptomatic carriers and vaccination classes makes the model complicated, emphasizing the silent role that asymptomatic people can play in disease transmission. across this study, we employ mathematical and graphical analysis to deconstruct the dynamic of HBV transmission across several demographic categories, including Susceptible, latent, acute infected with symptoms and asymptotically infected with no such visible symptoms, carriers, treatment, vaccination, and the recovered individuals. Our mathematical study reveals that the proposed model is both locally and globally stable when the basic reproduction number, $R_{0}$, falls below unity. Our model provides insightful estimates of how HBV will behave under various intervention approaches through simulation utilizing the (NSFD) technique. This research provides a solid foundation for public health organizations that invest heavily in the fight against hepatitis, such as the World Health Organization (WHO) and national public health agencies. These institutions can enhance how they limit community transmission by absorbing the insights obtained by our model, particularly in terms of asymptomatic carriers and the success of immunization efforts.
