# Correction: Global dynamics and computational modeling for analyzing and controlling Hepatitis B: A novel epidemic approach

**DOI:** 10.1371/journal.pone.0340134

**Published:** 2026-01-02

**Authors:** Muhammad Farhan, Zahir Shah, Zhi Ling, Kamal Shah, Thabet Abdeljawad, Saeed Islam, Hakim A. L. Garalleh

Two additional affiliations are missing for the fifth author. Thabet Abdeljawad is also affiliated with the Department of Mathematics and Applied Mathematics, School of Science and Technology, Sefako Makgatho Health Sciences University, Ga-Rankuwa, South Africa, and the Department of Medical Research, China Medical University, Taichung 40402, Taiwan.

There are errors in the Funding statement. The correct Funding statement is as follows: Authors are thankful to Prince Sultan University for APC and support through TAS research lab.

Additionally, the article is missing an Acknowledgements section. The Acknowledgements section should read as follows: Authors are thankful to Prince Sultan University for APC and support through TAS research lab.
